# Integrated Systems Analysis of the Murine and Human Pancreatic Cancer Glycomes Reveals a Tumor-Promoting Role for ST6GAL1

**DOI:** 10.1016/j.mcpro.2021.100160

**Published:** 2021-10-09

**Authors:** Emma Kurz, Shuhui Chen, Emily Vucic, Gillian Baptiste, Cynthia Loomis, Praveen Agrawal, Cristina Hajdu, Dafna Bar-Sagi, Lara K. Mahal

**Affiliations:** 1 Department of Cell Biology, NYU Grossman School of Medicine, New York, New York, USA; 2 Department of Chemistry, Biomedical Research Institute, New York University, New York, New York, USA; 3 Department of Biochemistry and Molecular Pharmacology, NYU Grossman School of Medicine, New York, New York, USA; 4 Department of Pathology, NYU Grossman School of Medicine, New York, New York, USA; 5 Office of Science and Research, NYU Grossman School of Medicine, New York, New York, USA

**Keywords:** lectin microarray, glycosylation, ST6GAL1, pancreatic cancer, lectin array, AAL, *Aleuria aurantia* lectin, AIA, *Artocarpus integrifolia* agglutinin, AOL, *Aspergillus oryzae* lectin, bulk-seq, bulk RNA sequencing, diCBM40, dimeric carbohydrate-binding module 40, DSA, *Datura stramonium* agglutinin, FFPE, formalin-fixed paraffin-embedded, FUT8, alpha-(1,6)-fucosyltranferase, GNA, *Galanthus nivalis* agglutinin, GRFT, *griffithsin*, H84T, H84T banana lectin, TJA-I, *Trichosanthes japonica* agglutinin, KC, p48^Cre^;LSL^KRASG12D^, LcH, *Lens* c*ulinaris*, LEA, *Lycopersicon esculentum* agglutinin, MAA, *Maackia amurensis* agglutinin, MAL-I, *Maackia amurensis* lectin-I, MGAT3, beta-1,4-mannosyl-glycoprotein 4-beta-N-acetylglucosaminyltransferase, MNA-G, *Morniga* G, MNA-M, *Morniga* M, MPA, *Maclura pomifera* agglutinin, NPA, *Narcissus pseudonarcissus* agglutinin, PanIN, pancreatic intraepithelial neoplasia, PC, principal component, PCA, principal component analysis, PDAC, pancreatic ductal adenocarcinoma, PHA-E, *Phaseolus vulgaris* lectin E, poly-LacNAc, poly-N-acetyllactosamine, PSA, *Pisum sativum* agglutinin, PSL, *Polyporus squamosus lectin*, SK1, *Streptococcus sanguinis SK1*, SLBR-H, siglec-like binding region *Streptococcus gordonii strains DL1*, SLBR-N, siglec-like binding region *Streptococcus gordonii strains UB10712*, SNA, *Sambucus nigra* agglutinin, ST3GAL1/2/3/4/5/6, ST3 beta-galactoside alpha-2,3-sialyltransferase 1/2/3/4/5/6, ST6GAL1/2, ST6 beta-galactoside alpha-2,6-sialyltransferase 1/2, ST6KC, ST6GAL1^flx/flx^;p48^Cre^;LSL^KRASG12D^, TMA, tissue microarray, WGA, wheat germ agglutinin

## Abstract

Pancreatic ductal adenocarcinoma (PDAC) is the third leading cause of cancer death in the United States. Glycans, such as carbohydrate antigen 19-9, are biomarkers of PDAC and are emerging as important modulators of cancer phenotypes. Herein, we used a systems-based approach integrating glycomic analysis of the well-established KC mouse, which models early events in transformation, and analysis of samples from human pancreatic cancer patients to identify glycans with potential roles in cancer formation. We observed both common and distinct patterns of glycosylation in pancreatic cancer across species. Common alterations included increased levels of α-2,3-sialic acid and α-2,6-sialic acid, bisecting GlcNAc and poly-N-acetyllactosamine. However, core fucose, which was increased in human PDAC, was not seen in the mouse, indicating that not all human glycomic changes are observed in the KC mouse model. *In silico* analysis of bulk and single-cell sequencing data identified ST6 beta-galactoside alpha-2,6-sialyltransferase 1, which underlies α-2,6-sialic acid, as overexpressed in human PDAC, concordant with histological data showing higher levels of this enzyme at the earliest stages. To test whether ST6 beta-galactoside alpha-2,6-sialyltransferase 1 promotes pancreatic cancer, we created a novel mouse in which a pancreas-specific genetic deletion of this enzyme overlays the KC mouse model. The analysis of our new model showed delayed cancer formation and a significant reduction in fibrosis. Our results highlight the importance of a strategic systems approach to identifying glycans whose functions can be modeled in mouse, a crucial step in the development of therapeutics targeting glycosylation in pancreatic cancer.

## Highlights

• Systems approach shows the glycome of KC mouse only partially overlays human PDAC.
• α2,6-Sialylation and ST6GAL1 are upregulated in mouse and human pancreatic cancer.
• Pancreas-specific ST6GAL1 KO in KC mouse delays cancer formation.
• Results show the importance of identifying glycans that can be modeled in mice.

## Graphical Abstract

**Figure:**
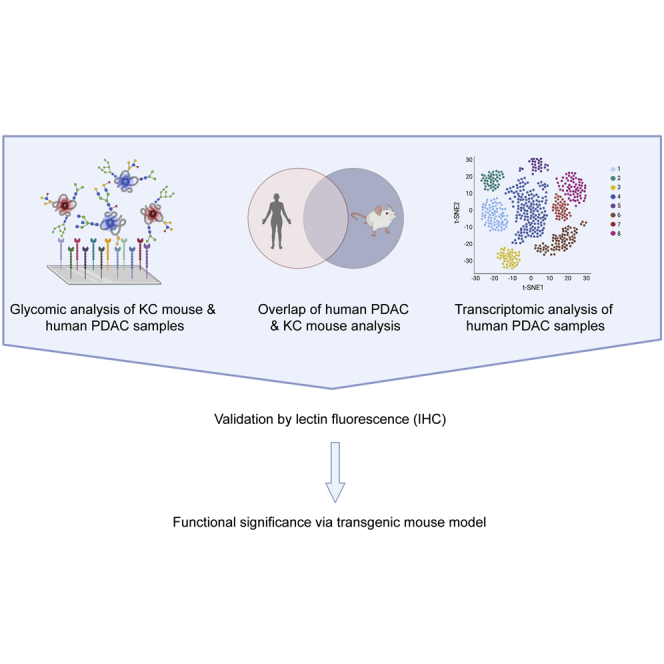

## In Brief

Pancreatic ductal adenocarcinoma is a leading cause of cancer death. Glycans are emerging as important modulators of cancer phenotypes. Herein, we used a strategic systems-based approach integrating glycomics of the KC mouse, modeling early events in transformation, with human data to identify ST6GAL1 (*e.g.*, α-2,6-sialic acid) as a potential cancer promoter. A pancreas-specific ST6GAL1 KO in the KC mouse delayed cancer formation and reduced fibrosis. Our results highlight the importance of identifying glycans whose functions can be modeled in mice.

The survival rate for pancreatic ductal adenocarcinoma (PDAC) beyond 5 years is very low. This disease is the third leading cause of cancer-related death in the United States, with few treatment options once malignant transformation has occurred. Growing evidence has identified altered glycosylation as a hallmark of solid tumor cancers (1). Glycosylation contributes to multiple facets of both cancer initiation (*e.g.*, sustained proliferative signaling, resistance to cell death, etc.) and progression (*e.g.*, invasion, metastasis, tumor-promoting inflammation, etc.) (1). Several studies have identified the α-2,3-sialylated glycans sialyl Lewis^A^, also known as carbohydrate antigen 19-9, and the sialylated antigen, sialylated tumor-related antigen, as biomarkers of pancreatic cancer and as prognostic indicators (2, 3, 4). Heterologous expression of human enzymes that can biosynthesize carbohydrate antigen 19-9 in a mouse model increased pancreatic inflammation, pointing to a potential role of this epitope in disease initiation (5). Pancreatic cell culture models have also shown potential roles for glycosylation in cell stemness, invasiveness, and drug resistance (6, 7, 8, 9).

Much of our knowledge of cancer biology comes from mouse models. Initial events in pancreatic cancer cannot currently be assessed using clinical samples, as 80% of cases are diagnosed at stage III or beyond (Seer.Cancer.gov). The KC mouse model (KC: p48^Cre^; LSL^KRASG12D^) is considered an accurate representation of the axis of early human disease (10). This model uses a mutant KR, as observed in 90% of the patients, to drive pancreatic transformation. Glycosylation has both conserved and species-specific components, which may impact the modeling of human PDAC in mice (11). Although many glycans and glycosylation enzymes are conserved across species, some can have distinct biological functions in different organisms. For example, mutations and deletions in ST3 beta-galactoside alpha-2,3-sialyltransferase (ST3GAL5), an enzyme that biosynthesizes the ganglioside GM3, are responsible for a spectrum of severe epileptic disorders in humans (12). However, a mouse model in which ST3GAL5 (also known as GM3 synthase) is knocked out does not show this phenotype (13, 14). The lack of direct conservation between the mouse and human biology when centered on the glycome is a crucial issue in modeling human disease.

**Scheme 1:**
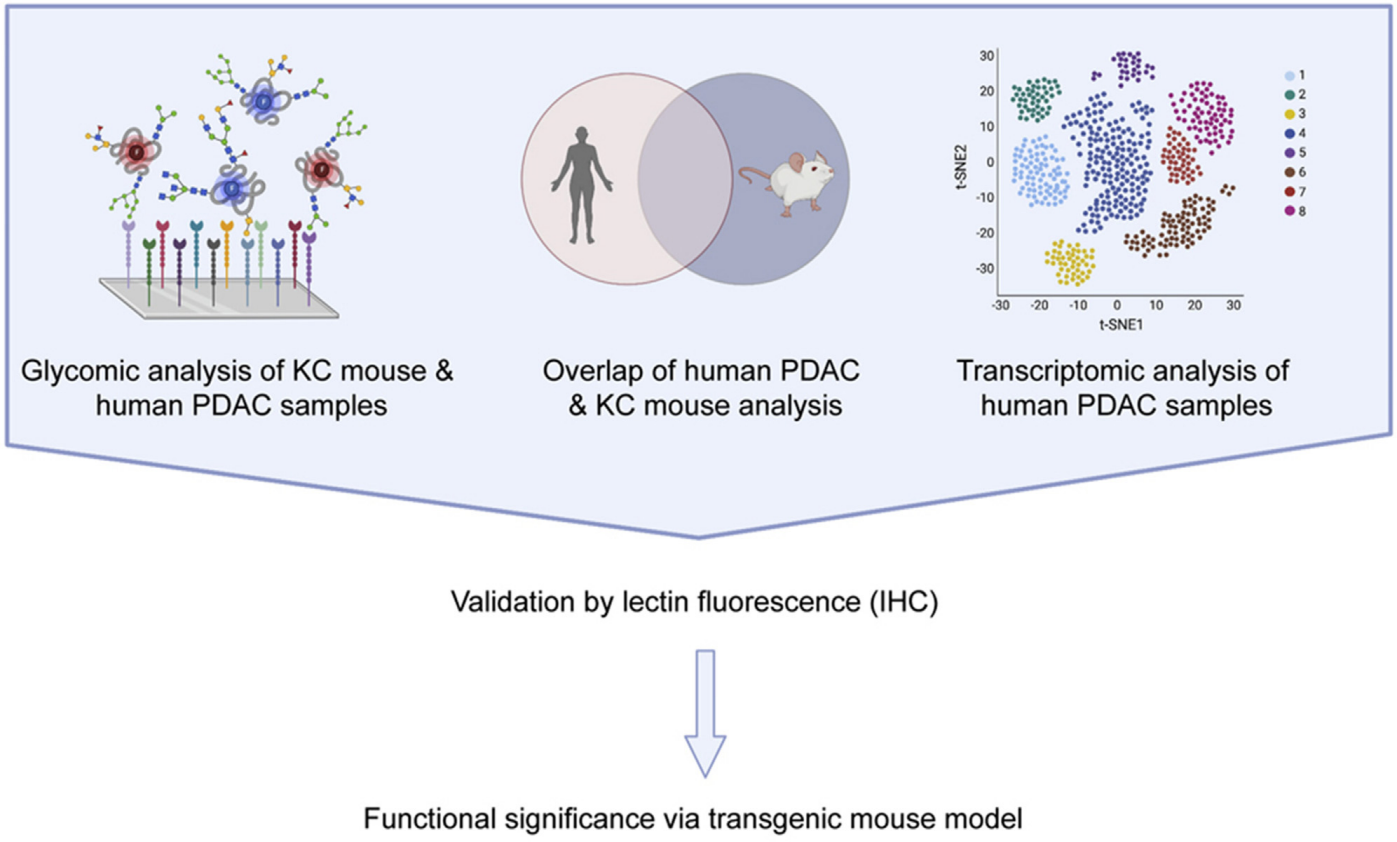
Overview of integrated systems-approach to understand the role of glycosylation in pancreatic cancer.

Herein, we use a systems-based approach integrating glycomic analysis of the KC mouse model with glycomic and transcriptomic data from human PDAC to identify and probe the functional significance of aberrant glycosylation in pancreatic cancer formation (Scheme 1). Specifically, we show that α-2,3- and α-2,6-sialic acids, bisecting GlcNAc and poly-N-acetyllactosamine (poly-LacNAc), are significantly upregulated in both the KC mouse model and human PDAC, whereas other glycans, such as core fucose, are only seen in one of the two sample sets. Using transcriptomic data, we identified specific glycosyltransferases driving these glycopatterns, narrowing our focus upon ST6 beta-galactoside alpha-2,6-sialyltransferase 1 (ST6GAL1), the main enzyme underlying α-2,6-sialic acid. *In silico* analysis of single-cell sequencing data pointed to an increase of ST6GAL1 in the cancerous ductal compartment. Multiplex immunofluorescence confirmed that ST6GAL1 protein is significantly upregulated in human disease, with elevated levels detected as early as stage I. To test whether this enzyme is a promoter of pancreatic cancer, we created a pancreas-specific genetic deletion of ST6GAL1 in the KC mouse model (ST6GAL1flx/flx;p48Cre; LSLKRASG12D [ST6KC]). The analysis of this model showed delayed cancer formation and a significant reduction in fibrosis. Our results highlight the importance of a strategic systems approach to identifying glycans whose functions can be modeled in mouse, a crucial step in the development of therapeutics targeting glycosylation in pancreatic cancer.

## Experimental Procedures

### Animals and Disease Models

C57BL/6 mice were purchased from the Jackson Laboratory and bred in-house. The KC mice, which express *Kras*^*G12D*^ in the progenitor cells of the pancreas, were bred in-house (15). The ST6KC mice were generated by crossing ST6GAL1^flx/flx^ mice (Jackson Laboratory) into the p48-Cre used to generate KC mice. The resultant mice were crossed with LSL-*KRas*^*G12D*^. The mice were confirmed by genotyping. Both male and female mice were used, and animals were age-matched within each experiment as indicated. All studies were reviewed by the Institutional Animal Care and Use Committee at NYU School of Medicine (IACUC #170513). The experiments were conducted in accordance with the NYU School of Medicine policies on the care, welfare, and treatment of laboratory animals.

### Human Sample Acquisition

All human pancreatic tumor and normal pancreas specimens were collected under an Institutional Review Board approved protocol (IRB#10-2519) utilizing REDCap 1253 within the Center for Biospecimen Research and Development at the NYU Langone Medical Center. The studies in this work abide by the Declaration of Helsinki principles. The patient characteristics are given in supplemental Table S1.

### Murine and Human Sample Processing for Lectin Array

Formalin-fixed, paraffin-embedded (FFPE) tissues from the mouse and human were prepared for our lectin microarrays and labeled with Alexa Fluor 555 as previously described (16). A pooled reference sample, labeled with Alexa Fluor 647, was created for each experiment. The details of sample preparation are given in supplemental Table S2.

### Lectin Microarray Printing, Hybridization, and Analysis

The lectin microarrays were printed as previously described (17). The print lists for the mouse and human lectin microarrays, including lectin sources, are given in supplemental Table S3. Equal amounts of sample and reference (5 μg) were hybridized on each array, and data analysis was performed as previously described (18). Lectins whose signal-to-noise ratio was <3 for more than one-third of the samples in an experiment were excluded from the analysis (annotated in supplemental Table S3). The additional experimental information can be found in supplemental Table S2.

### Multiplex Immunofluorescence, Immunohistochemistry, Image Acquisition, and Quantification of Murine KC Samples

For histological analysis, murine KC pancreatic tissues were fixed with 10% buffered formalin, dehydrated in ethanol, and embedded with paraffin (FFPE). 5 μm sections of FFPE murine KC were mounted on slides. For immunohistochemical staining, briefly, slides were deparaffinized and stained with H&E (S3301, Dako) or Gomori trichrome followed by whole-tissue scanning at 40× magnification on an Aperio AT2 (Leica Biosystems). The preserved acinar area of the KC pancreatic tissues was quantified using Photoshop software (Adobe Acrobat) and calculated as a fraction of the total pixels of the pancreatic tissue. Percent fibrosis was quantified using the total blue positive pixels in Photoshop software (Adobe Acrobat), which is determined as a fraction of the total pixels of the pancreatic tissue.

### Human Tissue Microarray Samples

All staining of human tissues was performed on a Leica Bond RX automated stainer (Leica Microsystems). To ensure lectin specificity, *Sambucus nigra* agglutinin (SNA) conjugated to Cy3 (1:300 dilution, Vector Laboratories, cat # CL-1303) was incubated with 0, 100, or 200 mM of beta-D-lactose (Alfa Aesar, cat # H54447) in PBS, 0.1% Tween for 2 h at 22 °C. The liver sections stained with SNA in the absence of beta-D-lactose yielded strong signals. In contrast, SNA staining was blocked by both 100 and 200 mM beta-D-lactose. Before duplex staining, BioMax PA2072a tissue microarray (TMA) underwent deparaffinization and heat retrieval with Bond ER2 buffer (Leica Microsystems, cat # AR9640). The slide was then incubated with anti-human ST6GAL1 (Proteintech, cat # 14355-1-AP) followed by Opal Polymer horseradish peroxidase Ms + Rb (Akoya Biosciences, cat # ARH1001EA) and tyramide-linked 650 Opal fluorophore (Akoya Biosciences, cat # FP1496001KT) to amplify signals. The antibody complexes were stripped from the tissues with the ER2 buffer, leaving the fluorophore covalently attached to ST6GAL1, and the TMA subsequently stained with the SNA-cy3 lectin. Image acquisition was performed on a Vectra Polaris multispectral imaging system (Akoya Biosciences). The fluorophores were spectrally unmixed using inForm software (Akoya Biosciences), and the images were exported as tif files for scoring. Cell-type identification (epithelial *versus* stromal) and quantification of the number of ST6GAL1- or SNA-positive cells per high-power field were performed by an independent pathologist and validated by a second scorer for each core.

### Human Tissue RNA Expression Profiling

PDAC tumor and adjacent normal tissue mRNA raw expression profiles were downloaded from the Gene Expression Omnibus [accession #: GSE16515 and GSE15471] and normalized in one batch using a GC-content background-corrected Robust Multi-array Average algorithm in R, a language and environment for statistical computing, as previously described (19). Hierarchical clustering was performed in GENE-E (https://software.broadinstitute.org/GENE-E/index.html), and adjacent normal tissues clustering with PDAC tumors and PDAC tumors clustering with adjacent normal tissues were removed. The patients with expression profiles containing the gene of interest in both PDAC and adjacent normal groups were used for differential expression analysis.

### Cluster Identification and Single-Cell Sequencing Analysis

Publicly available raw single-cell sequencing patient data were obtained (20). A quality control was then performed on the cells to calculate the number of genes and the proportion of mitochondrial genes for each cell using iCellR R package (v0.99.0) (https://github.com/rezakj/iCellR), and the cells with the low number of covered genes (gene count < 500) and high mitochondrial counts (mitochondrial genes > 0.1) were filtered out. Matrix normalization, geometric library size factor normalization, and gene statistics analysis were performed, as previously published (21). Briefly, genes with high coverage (top 500) and high dispersion (dispersion >1.5) were chosen and principal component analysis (PCA) was performed, a second round of PCA was performed based on the top 20 and bottom 20 genes predicted in the first ten dimensions of PCA to fine-tune the results, clustering was performed (iCellR options; clust.method = “kmeans,” dist.method = “Euclidean,” and index.method = “silhouette”) on principal components (PCs) with high standard deviation (top ten PCs), and Uniform Manifold Approximation and Projection was performed on the top ten PCs. Marker genes for each cluster were determined based on the fold change and adjusted *p*-value (*t* test), and average gene expression for each cluster was calculated using iCellR.

### Statistical Analysis and The Cancer Genome Atlas

The data are presented as the mean ± standard error. The data on gene expression and survival in human tissues were derived from The Cancer Genome Atlas utilizing the cBIOportal (https://www.cbioportal.org/). Statistical significance was determined by the Student’s *t* test and the Wilcoxon test using GraphPad Prism 7 (GraphPad Software). *p*-values <0.05 were considered significant.

### Experimental Design and Statistical Rationale

For glycomic analysis of the KC mouse model, n = 4 to 5 animals were used for each gender at each timepoint. For glycomic analysis of human PDAC, n = 12 PDAC and 12 normal samples were analyzed; however, two PDAC samples and one normal sample were sourced from the same patient. Thus, the dataset represents n = 10 unique PDAC and n = 11 unique normal patients. For more details, see supplemental Table S1. Transcriptomic analysis was as described above. Multiplex immunohistochemistry was performed on TMAs containing triplicates of unique cores from n = 60 patients with PDAC and nine normal patients, for a total of n = 180 PDAC and 27 normal cores. Two PDAC cores were removed from the analysis because of quality control concerns. For more details, see (https://www.biomax.us/tissue-arrays/Pancreas/PA2072a). For the analysis of ST6KC and KC mice, n = 5 mice were used per group. Statistical analysis for each glycomic, transcriptomic, and histochemical analysis is described in the associated section.

## Results

### Glycomic Analysis of the KC Mouse Model Reveals Sex-Dependent Changes in the Glycome

**Fig. 1:**
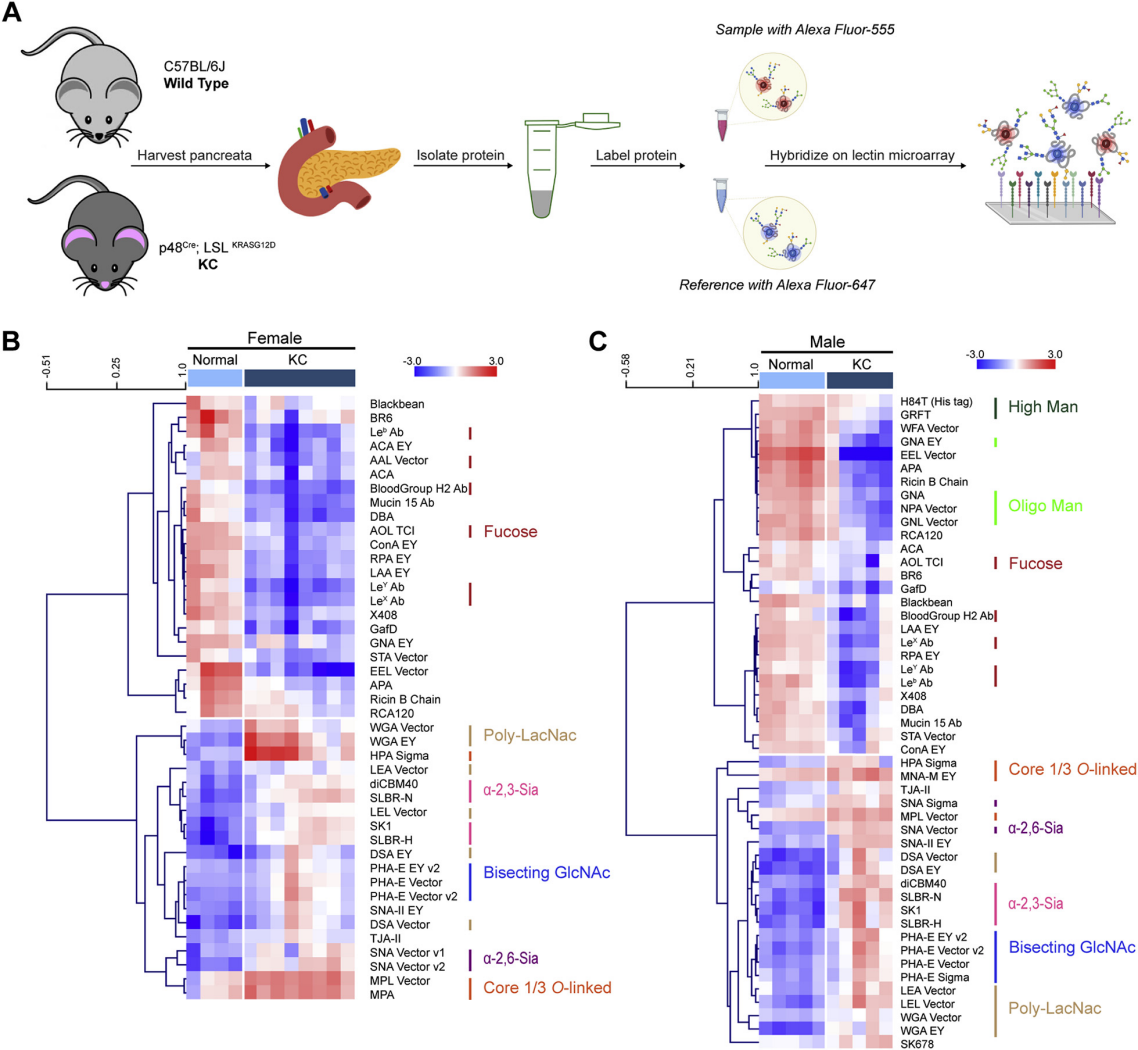
**Glycome of the pancreas from male and female KC mice differs from the normal pancreas.***A*, workflow of sample preparations for the dual-color lectin microarray analysis in KC mice. Glycoproteins were isolated from the KC mouse or normal mouse pancreata and labeled with Alexa Fluor 555-NHS. A pooled reference was orthogonally labeled with Alexa Fluor 647-NHS. Equal amounts of the sample and reference were mixed and hybridized on lectin microarrays (>100 probes). *B* and *C*, heat map of lectin microarray data for significant lectins. Significance was determined using the Student’s *t* test (*two-tailed*). Only lectins with *p* ≤ 0.05 were included in the heat map. The complete heat maps are shown in supplemental Fig. S1. The samples were organized manually (normal samples: *light blue*; KC samples: *dark blue*). The lectins were hierarchically clustered using Pearson correlation coefficient and average-linkage analysis. The median-normalized log_2_ ratios (sample (S)/reference(R)) of the female (*B*) and male (*C*) KC pancreas *versus* normal pancreas (14 weeks: normal, n = 4, KC, n = 8 for female; normal, n = 5, KC, n = 8 for male mice). *Red*, log_2_(S) > log_2_(R); *blue*, log_2_(R) > log_2_(S). The lectins binding *α*-2,3-sialosides (*pink*), *α*-2,6-sialosides (*purple*), bisecting GlcNAc (*navy*), N-acetyl-D-lactosamine (*brown*), oligo-mannose (*bright green*), high-mannose (*forest green*), core fucose (*charcoal*), *O*-linked glycans (*orange*), and fucose (*red*) are highlighted to the *right* of the heat map.

To assess the functional impact of glycosylation on pancreatic cancer development and progression, we turned to the KC mouse model. We analyzed pancreata from genetically engineered KC mice bearing a p48-specific activating KRAS mutation (KC: p48^Cre^; LSL^KRASG12D^). These mice develop dysplasia, pancreatic intraepithelial neoplasia (PanIN) lesions, fibrosis, and eventually advanced cancer of the pancreas (10). Pancreata were harvested from 14-week-old female (n= 8) and male (n = 5) KC mice, along with appropriately age- and gender-matched littermate controls (normal: female, n = 4; male, n= 5). By this timepoint, the significant levels of advanced PanIN lesions and displacement of normal acinar tissue are observed. FFPE pancreatic tissue samples were processed and analyzed using our dual-color lectin microarray technology (16). The lectin microarrays use carbohydrate-binding proteins with well-defined specificities to detect glycan changes between samples and have been used for cancer glycomics (16, 22, 23, 24). Heat maps of lectins showing significant differences in binding between the KC pancreas and normal tissue, separated by sex, are shown in Figure 1. The heat maps of all lectins and volcano plots identifying significant lectins are shown in supplemental Figs. S1–S3.

Glycomic analysis shows gender-dependent and gender-independent differences in pancreatic cancer in mice. Focusing on common differences between sexes, we observe increases in sialic acids (2- to 4-fold) in the KC mouse pancreata. These changes were seen in α-2,6-sialosides (lectins: SNA) and α-2,3-sialosides (lectins: dimeric carbohydrate-binding module 40 [diCBM40] (25), siglec-like binding region Streptococcus gordonii strains DL1 [SLBR-H], siglec-like binding region Streptococcus gordonii strains UB10712 [SLBR-N] (26, 27), and siglec-like binding region *Streptococcus* sanguinis strains [SK1]). Similar dramatic increases were also observed in bisecting GlcNAc (Phaseolus vulgaris lectin E [PHA-E]) and poly-*N*-Acetyl-D-lactosamine (Datura stramonium agglutinin [DSA], Lycopersicon esculentum agglutinin [LEA], and wheat germ agglutinin [WGA]). In addition, we observed increases in core 1/3 *O*-linked glycans (*Morniga* M [MNA-M] agglutinin, *Morniga* G [MNA-G,] agglutinin, Maclura pomifera agglutinin [MPA], and *Artocarpus integrifolia* agglutinin [AIA]). In contrast, the overall fucose levels decrease in the KC mice relative to normal controls (*Aspergillus oryzae* lectin [AOL] and *Aleuria aurantia* Lectin [AAL]). In male mice, we also observe decreasing levels of high-mannose (Man_7–9_, lectins: H84T and *griffithsin* [GRFT]) and oligo-mannose (Man_5–7_, lectins: Galanthus nivalis agglutinin [GNA] and *Narcissus pseudonarcissus* agglutinin [NPA]). This is not observed in the female cohort.

### Temporal Dynamics of Glycomic Changes in the KC Mouse Model

**Fig. 2:**
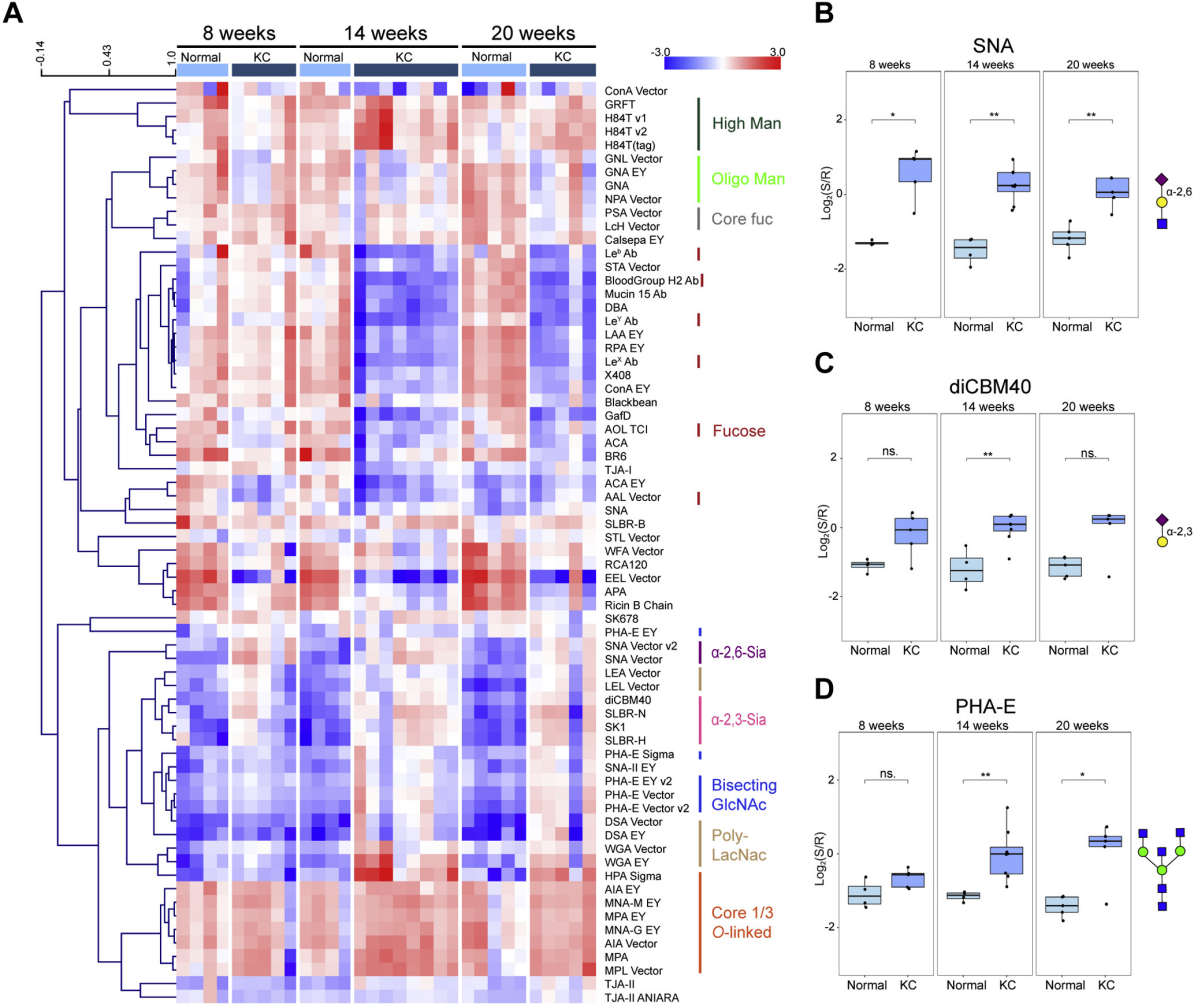
**Time-course of the pancreas glycome from female KC mice.***A*, heat map of the lectin microarray data. The median-normalized log_2_ ratios (sample (S)/reference(R)) of the pancreatic samples were ordered by timepoints (8 weeks: normal (*light blue*), n = 5, KC (*dark blue*), n = 5; 14 weeks: normal, n = 4, KC, n = 8; 24 weeks: normal, n = 5, KC, n = 5). The lectins were hierarchically clustered using the Pearson correlation coefficient and average-linkage analysis. *Red*, log_2_(S) > log_2_(R); *blue*, log_2_(R) > log_2_(S). The lectins binding *α*-2,3-sialosides (*pink*), *α*-2,6-sialosides (*purple*), bisecting GlcNAc (*navy*), N-acetyl-D-lactosamine (*brown*), oligo-mannose (*bright green*), high-mannose (*forest green*), core fucose (*charcoal*), *O*-linked glycans (*orange*), and fucose (*red*) are highlighted to the right of the heat map. *B*, time-course analysis of *α*-2,6-sialosides by SNA (data for the SNA vector are shown). *C*, time-course analysis of *α*-2,3-sialosides by diCBM40. *D*, time-course analysis of bisecting GlcNAc by PHA-E as a function of timepoints (data for PHA-E vector v2 are shown). ∗*p* < 0.05; ∗∗*p* < 0.01; Wilcoxon’s *t* test. The glycans bound by lectins are shown in the Symbolic Nomenclature for Glycomics (SNFG) at the side of the boxplots (47). The data for the male mice are shown in supplemental Fig. S3. diCBM40, dimeric carbohydrate-binding module 40; ns, not statistical; PHA-E, *Phaseolus vulgaris* lectin E; SNA, *Sambucus nigra* agglutinin.

In our KC model, the 14-week timepoint has both early dysplastic lesions and more advanced cancerous lesions. To examine whether our glycomic signature is a hallmark of transformation or of progression, we examined an earlier timepoint (6–8 weeks) and a later timepoint (20 weeks). By 6 to 8 weeks, the KC model shows the first significant evidence of pancreatic dysplasia by histology. At 20 weeks, the severity and frequency of PanIN lesions have increased, and fibrotic deposition can be observed. Pancreata from the KC mice were harvested at 8 and 20 weeks of life and compared with age- and gender-matched controls using our microarray technology, as previously described. For simplicity, the heat map for the female KC mice is shown in Figure 2 and the heat map for the male KC mice is shown in supplemental Fig. S4.

Although the predominant glycan signature of the pancreas did not change with age in normal mice, we did observe a few alterations. The levels of core 1/3 *O*-glycans (MPA, MNA-G, and AIA, Fig. 2*A*) decreased with age in the normal pancreas, but not in the KC mice, making the increase observed with cancer at 14 weeks more prominent by 20 weeks of age. In contrast, we saw no changes in the normal levels of N-glycans with age. The relative increases of α-2,6-sialosides (SNA, Fig. 2, *A* and *B* and supplemental Fig. S4, *A* and *B*) and α-2,3-sialosides (diCBM40, SLBR-H, SLBR-N, and siglec-like binding region *Streptococcus sanguinis strains SK1*, Fig. 2, *A* and *C* and supplemental Fig. S4, *A* and *C*) in the KC mice were observed by 8 weeks and conserved across all timepoints (14 and 20 weeks). We saw similar temporal changes in bisecting GlcNAc (PHA-E, Fig. 2, *A* and *D* and supplemental Fig. S4, *A* and *D*) and poly-LacNAc levels (DSA, LEA, and WGA, Fig. 2*A* and supplemental Fig. S4*A*). The timing of glycan expression with pancreatic cancer development indicates that these glycans may play roles in both the onset and progression of disease.

### Human Pancreatic Cancer Glycome Overlaps Select Glycomic Changes in the KC Mouse

**Fig. 3:**
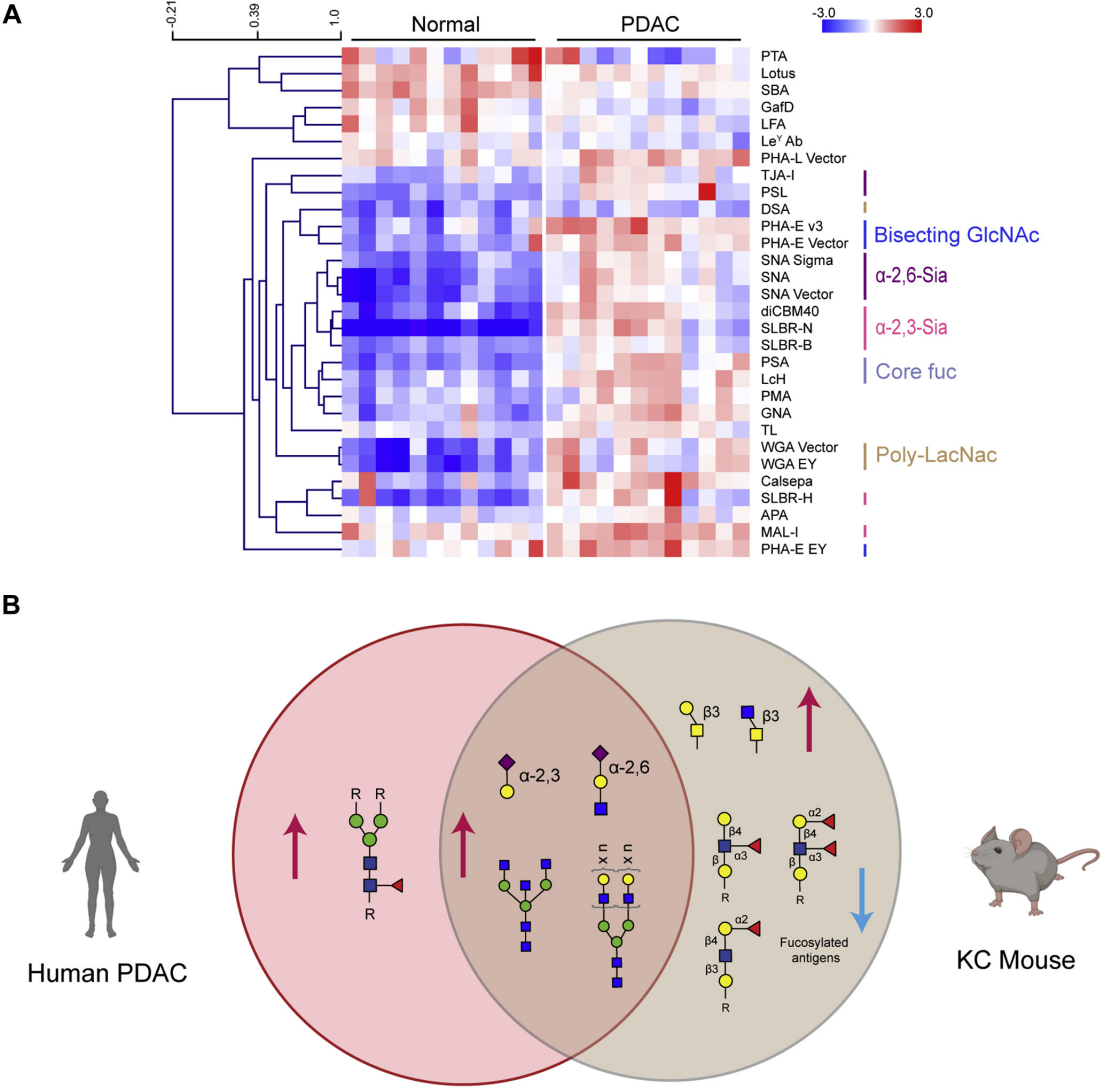
**Overlap of the glycomic analysis of the human PDAC and KC mouse.***A*, heat map presenting statistically significant lectins (*p* < 0.05, Student’s *t* test (*two-tailed*)) from lectin microarray analysis of the human samples is shown. The samples were organized manually. The lectins were hierarchically clustered using the Pearson correlation coefficient and average-linkage analysis. The median-normalized log_2_ ratios (sample (S)/reference(R)) were ordered by the sample type (normal, n = 12 and PDAC, n = 12). *Red*, log_2_(S) > log_2_(R); *blue*, log_2_(R) > log_2_(S). *B*, Venn diagram showing the overlap between significant glycan epitopes in both the human PDAC samples and female KC mouse samples at 14 weeks. PDAC, pancreatic ductal adenocarcinoma; poly-LacNAc, poly-N-acetyllactosamine.

To study whether pancreatic cancer displays an altered glycome from the normal pancreas, we used our dual-color lectin microarray technology (supplemental Fig. S5*A*) (17, 28). We obtained 12 biopsy samples from noncancerous pancreata and 12 from patients with PDAC (supplemental Table S1). The previous glycomic analysis on pancreatic cancer used “normal adjacent” tissue, but this is not considered representative of the nontransformed pancreas (29, 30). FFPE pancreatic tissue samples were solubilized and analyzed, as previously described (supplemental Table S2) (16). The heat map of lectins showing significant differences in binding between pancreatic cancer and normal tissue is shown in Figure 3*A*. The complete heat map and associated volcano plot are shown in supplemental Fig. S5, *B* and *C*.

We observed strong differences between the noncancerous and cancerous pancreatic glycome, with greater than one-third of all lectins on the array showing statistically significant differences in binding. In PDAC, we observed significant increases in sialic acids, with changes in both α-2,6-sialosides (lectins: SNA, *Trichosanthes japonica* agglutinin [TJA-I], and *Polyporus squamosus* lectin [PSL], average increase: ∼2.7-fold) and α-2,3-sialosides (diCBM40, SLBR-H, SLBR-N, *Maackia amurensis* lectin-I [MAL-I], and *Maackia amurensis* agglutinin [MAA], ∼3.4-fold). Core fucosylation (*Pisum sativum* agglutinin [PSA] and *Lens culinaris* [LcH], ∼2.7-fold), bisecting GlcNAc (PHA-E, ∼2.2-fold) and poly-LacNAc (WGA, DSA, and LEA, ∼2.1-fold), also increased relative to normal. We did not observe any significant differences in sialyl Lewis^A^. This is consistent with the literature reports of heterogenous expression of this serum biomarker in the solid tumor (31).

Only a subset of the overall glycan signature observed in the human PDAC data was represented in the KC mouse model (Fig. 3*B*). Conserved glycomic changes between the two species include α-2,6-sialosides and α-2,3-sialosides, bisecting GlcNAc and poly-LacNAc. The core fucosylation signature, which is a strong signature in the human PDAC, was not observed in the KC mouse model. Conversely, the increase in core 1/3 *O*-glycans and decrease in the overall fucosylation that was clear in the KC mouse were not seen in the human data. In the subsequent analysis, we then focused on glycosylation epitopes conserved between species.

### Bulk Transcriptomic Analysis and Single-Cell Sequencing Reveal ST6GAL1 Is Enriched in Cancerous Ducts

**Fig. 4:**
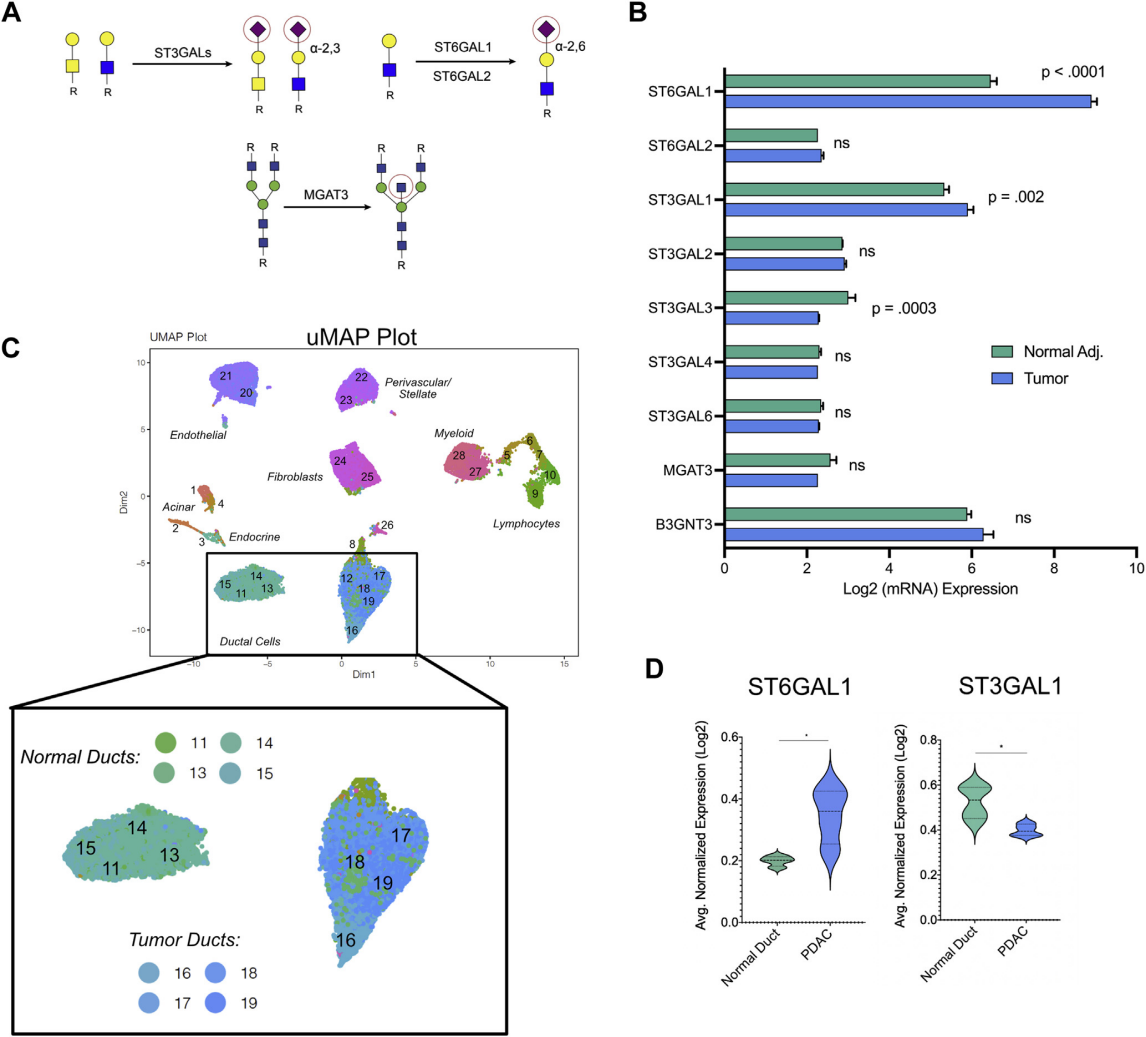
**Transcriptomic analysis identifies ST6GAL1 and ST3GAL3 as enriched in the cancerous ducts.***A*, biosynthetic pathways for glycans underlying select lectin signatures are shown. ST3GALs are responsible for transferring α-2,3-sialosides and ST6GAL1/2 for α-2,6-sialosides, and MGAT3 for bisecting GlcNAc. The glycans are annotated following the Symbolic Nomenclature for Glycans. *B*, transcriptomic analysis assessing the mRNA levels of select glycosyltransferases between normal adjacent and matched cancerous tissues within the same patient. *C*, uMAP plots representing the cells isolated from patients with PDAC (n = 24) and normal pancreata (n = 11) pooled on single-cell sequencing. The clusters representing the normal ductal cells and tumor ductal cells are highlighted. *D*, violin plot showing the comparison of ST6Gal1 levels in normal (*green*) *versus* PDAC (*blue*) ductal clusters. The clusters in each group are combined. The violin plots showing the comparison of ST6Gal1 and ST3GAL1 levels in normal (*green*) *versus* PDAC (*blue*) ductal clusters. The clusters in each group are combined. ns, *p* > 0.05; ∗*p* < 0.05; student’s *t* test (*two-tailed*). MGAT3, beta-1,4-mannosyl-glycoprotein 4-beta-N-acetylglucosaminyltransferase; ns, not statistical; PDAC, pancreatic ductal adenocarcinoma; ST3GAL3, ST3 beta-galactoside alpha-2,3-sialyltransferase 3; ST6GAL1, ST6 beta-galactoside alpha-2,6-sialyltransferase 1; uMAP, Uniform Manifold Approximation and Projection.

We next sought to determine glycosyltransferases that underlie the observed glycomic signatures. Using publicly available bulk RNA sequencing (bulk-seq) data from three separate studies performed on the same platform, we interrogated the expression levels of select biosynthetic enzymes (ST6GAL1, ST6GAL2 [α-2,6-sialylation], ST3GAL1, ST3GAL2, ST3GAL3, ST3GAL4, ST3GAL6 [α-2,3-sialyation], beta-1,4-mannosyl-glycoprotein 4-beta-N-acetylglucosaminyltransferase [MGAT3] [bisecting GlcNAc], and B3GNT3 [transfers GlcNAc onto Gal forming poly-LacNAc], Fig. 4, *A* and *B*). The comparison of human pancreatic cancer to the adjacent normal pancreas showed statistically significant increases for ST6GAL1 and ST3GAL1 in PDAC. The PDAC tumor and its microenvironment comprise many cell types, including ductal cells, immune cells, fibroblasts, and endothelia. We analyzed a separate cohort of publicly available single-cell RNA sequencing data using iCellR to study cell type–specific expression of genes from our bulk-seq analysis, initially focusing on upregulated targets (ST6GAL1 and ST3GAL1) (20, 32, 33). Uniform Manifold Approximation and Projection clusters were defined by transcriptomic signatures typical of intrapancreatic cell types (Fig. 4*C* and supplemental Fig. S6). PDAC originates from exocrine tissue specifically composed of acinar and ductal cells. Strong ST6GAL1 expression was observed in endothelial cells, immune cells, and the cancerous ductal clusters (Fig. 4*D*). In addition, ST6GAL1 levels were associated with reduced survival (supplemental Fig. S7). The α-2,3-sialyltransferase ST3GAL1 was also observed in many cell types; however, it was not enriched in cancer-specific ductal cells (Fig. 4*D* and supplemental Fig. S8). Expanding our single-cell analysis identified several more candidate glycogenes that were significantly upregulated in the cancerous ducts (ST3GAL4, B3GNT3, alpha-(1,6)-fucosyltranferase [FUT8], and MGAT3), corroborating our lectin signature (supplemental Fig. S8). These candidates were not significant in the bulk-seq analysis; thus, we focused our attention on ST6GAL1, which was consistent throughout the different datasets. Our data suggest that α-2,6-sialic acid may be important for the formation and/or progression of cancerous ducts.

### ST6GAL1 Protein and α-2,6-Sialic Acid Levels Are Upregulated in Human Pancreatic Cancer

**Fig. 5:**
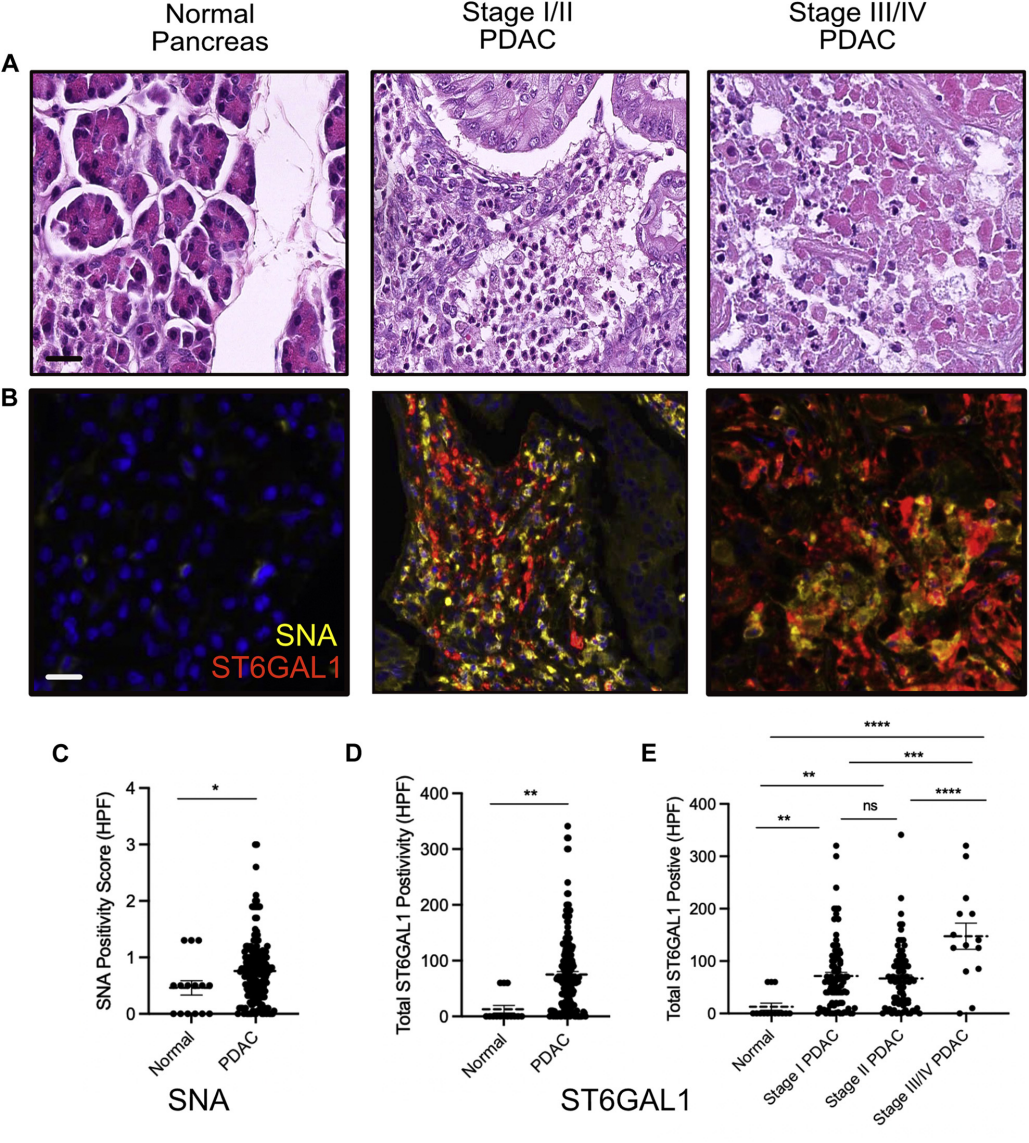
**Profiling of ST6GAL1 and SNA in human pancreatic cancer shows association with the stage and survival.***A*, H&E of the normal pancreas (*left*), stage I pancreatic adenocarcinoma (*center*), and stage IV PDAC (*right*) stained from a BioMax human tissue microarray. *B*, multiplex OPAL IF staining of SNA (*yellow*), ST6GAL1 (*red*), and DAPI (*blue*) on the corresponding normal pancreas and stage I and stage IV pancreatic adenocarcinoma purchased from BioMax human tissue microarray. The scale bars represent 25 μm. *C*, quantification of SNA-positive cells per high-powered field based on multiplex IF in normal *versus* all cancerous cases in human tissue microarray. Owing to the high number of positive cells, each HPF was given a score (1–3) based on the number of SNA-positive cells per field. *D*, quantification of ST6GAL1-positive cells per high-powered field in normal *versus* all cancerous cases in human tissue microarray. *E*, distribution of the total ST6GAL1-positive cells per high-powered field across normal and each stage of pancreatic cancer based on multiplex IF imaging of human pancreatic cancer tissue microarray. Statistical significance was evaluated using the Student’s *t* test (*two-tailed*): ns, *p* > 0.05; ∗*p* < 0.05; ∗∗*p* < 0.01; ∗∗∗*p* < 0.001; ∗∗∗∗*p* < 0.0001. HFP, high-powered field; ns, not statistical; PDAC, pancreatic ductal adenocarcinoma; SNA, *Sambucus nigra* agglutinin; ST6GAL1, ST6 beta-galactoside alpha-2,6-sialyltransferase 1.

To look more deeply at α-2,6-sialylation in human pancreatic cancer, we stained an orthogonal cohort of human PDAC tumors and normal pancreata for the ST6GAL1 protein and α-2,6-sialylation (SNA, Fig. 5 and supplemental Fig. S9). Consistent with both our lectin microarray data and transcriptomic analysis, we observed that α-2,6-sialic acid levels (as determined by SNA) and corresponding ST6GAL1 levels are both significantly upregulated in human pancreatic cancer (Fig. 5, *A*–*D*). Further analysis revealed that changes in ST6GAL1 could be observed at the earliest diagnosable stage of disease (Fig. 5*E*). Upon closer examination of the stained tissues, we observed ST6GAL1 in both stromal infiltrates and transformed epithelial cells (supplemental Fig. S9, *D* and *E*). Epithelial cells encompass endocrine, acinar, and ductal cells in the pancreas. Consistent with the single-cell sequencing analysis, epithelial expression of ST6GAL1 correlates more significantly with the disease state than stromal expression (supplemental Fig. S9, *D* and *E*). These data validate our previous analysis, implying a functional role for this enzyme in pancreatic cancer.

### Pancreas-Specific Deletion of ST6GAL1 in the KC Mouse Slows Cancer Development and Progression

**Fig. 6:**
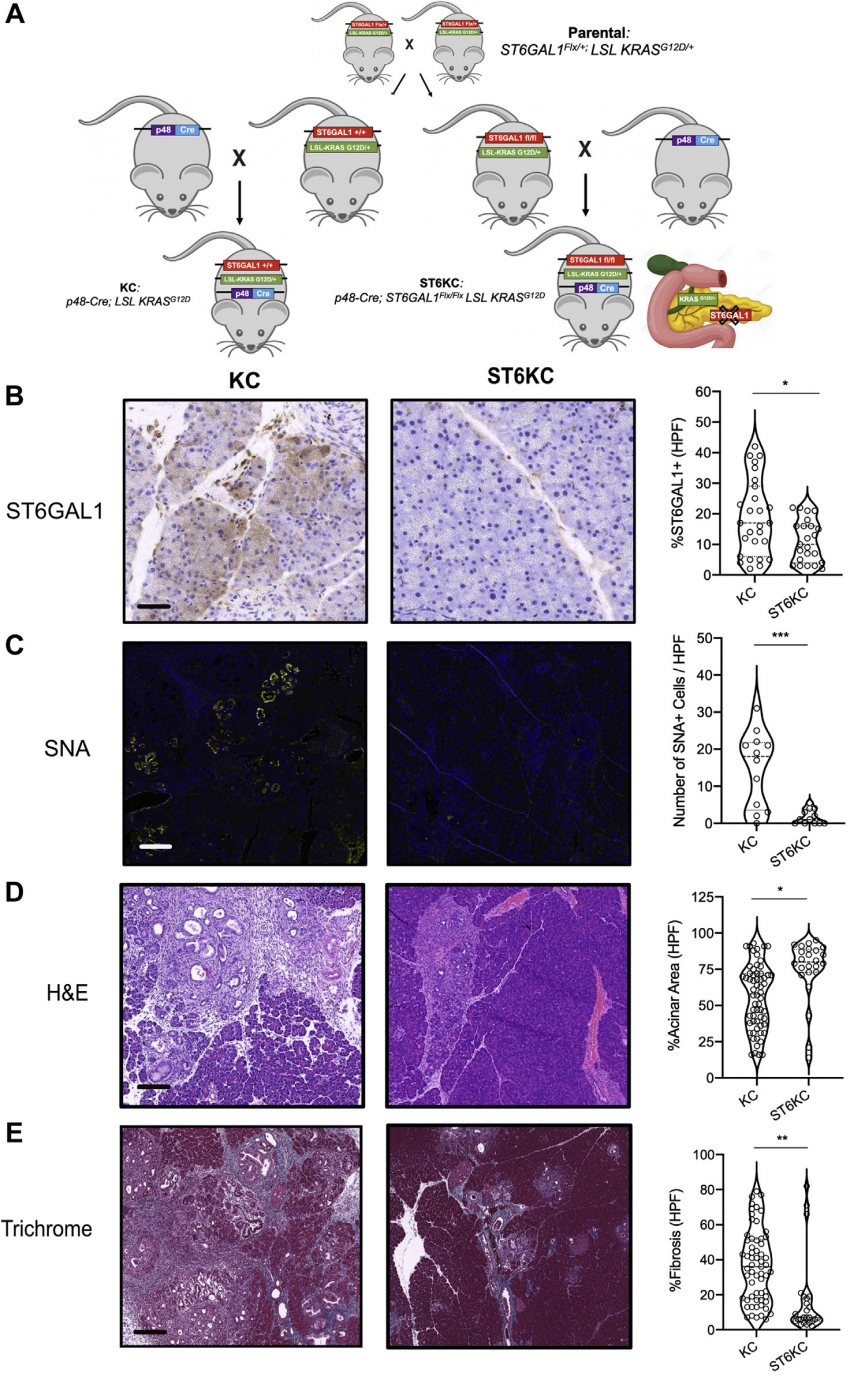
**Pancreas-specific deletion of ST6GAL1 reduces disease burden in murine PDAC.***A*, breeding schematic illustrating the generation of novel ST6KC mice. The offspring of parental strains crossed into p48-Cre mice drive the induction of mutant KRAS^G12D^ and deletion of ST6GAL1 under the same promoter. *B*, IHC staining for ST6GAL1 (*brown*) in KC and ST6KC mice (n = 3 per group). The number of ST6GAL1+ cells per high-powered field is quantified. The scale bar represents 50 μm. *C*, IF staining for SNA (*yellow*) and DAPI (*blue*) in KC and ST6KC mice (n = 3 per group). The number of SNA+ cells per high-powered field is quantified. The scale bar represents 100 μm. *D*, H&E of 14-week-old FFPE pancreata from KC and ST6KC mice (n = 5 per group). The percent of the preserved normal pancreas area is quantified per high-powered field. The scale bar represents 200 μm. *E*, trichrome and gomori (*blue*) stain of FFPE pancreata from 14-week-old KC and ST6KC mice (n = 5 per group). The percent of collagen deposition fibrosis is quantified per high-powered field on the right. The scale bar represents 200 μm. ∗*p* < 0.05; ∗∗*p* < 0.01; ∗∗∗*p* < 0.001; Student’s *t* test (*two-tailed*). FFPE, formalin-fixed paraffin-embedded; IF, immunofluorescence; IHC, immunohistochemical; PDAC, pancreatic ductal adenocarcinoma; SNA, *Sambucus nigra* agglutinin; ST6GAL1, ST6 beta-galactoside alpha-2,6-sialyltransferase 1; ST6KC, ST6GAL1^flx/flx^;p48^Cre^; LSL^KRASG12D^.

To determine whether ST6GAL1 plays a role in the development or progression of pancreatic cancer, we generated a novel ST6GAL1^flx/flx^ KC mouse (ST6KC). In these mice, a pancreatic lineage–specific p48-Cre drives both the expression of mutant KRASG12D and KO of ST6GAL1 (Fig. 6*A*). To validate the ST6GAL1 KO, we performed staining for ST6GAL1 in pancreata from the ST6KC mice at 14 weeks of age. As expected, we observed a significant loss of both ST6GAL1 protein and concomitant α-2,6-sialylation (as seen by SNA staining) in the ductal and acinar cells (Fig. 6, *B* and *C*). We next examined whether the KO altered the histological profile of pancreata from ST6KC as compared with the KC mice. We observed significant preservation of the normal acinar area in the ST6KC compared with the KC mice (Fig. 6*D*, *p* < 0.01). Examination of fibrosis, as quantified by trichrome and gomori staining, showed a significant reduction in the ST6KC mice (Fig. 6*E*, *p* < 0.01). Fibrosis is a hallmark of pancreatic cancer formation and severity. It is known to contribute to disease progression, immune suppression, and resistance to therapeutic interventions in both mouse and human diseases (34). Overall, our model demonstrates that targeted deletion of ST6GAL1 has a clear protective effect against pancreatic cancer formation and progression.

## Discussion

Pancreatic cancer is one of the deadliest cancers and is predicted to be the second leading cause of cancer-related deaths by 2030 (35). Although the incidence rate of pancreatic cancer has increased, the 5-year survival rate remains low, with few long-term survivors and high rates of therapeutic resistance (36). Long known as a hallmark of cancer, changes in glycosylation are emerging as crucial modulators of the cancer phenotype. Herein, we applied a strategic systems-based approach integrating lectin microarray analysis of mouse models, human samples, and *in silico* analysis of both bulk and single-cell sequencing data to investigate the role of glycosylation in promoting pancreatic cancer. Our approach guided our modeling of the impact of glycosylation on pancreatic cancer, enabling the testing of ST6GAL1 in the KC mouse model and identifying it as a promoter of this disease.

The full axis of pancreatic cancer progression from initiating events to metastatic spread is considered to be accurately modeled by the KC mouse (10). However, to date, no comparisons have been made between the glycome of this model and human pancreatic cancer. Reconciling common changes within these systems is critical to modeling the impact of the glycome on cancer development and progression. We observed both common and disparate glycan signatures in the two systems. Both the mouse and human showed prominent increases in α-2,3-sialosides and α-2,6-sialosides, bisecting GlcNAc and poly-LacNAc epitopes. These changes have been previously observed in several glycomic studies of human pancreatic cancer tissues (4, 29). Human PDAC shows an increase in the core fucose, an epitope associated with metastasis in several cancers (16, 37, 38, 39), which is not recapitulated in the KC mouse. This may be accounted for by the fact that at 20 weeks, the KC model does not exhibit metastatic spread. In contrast, the mouse model showed a loss of complex fucose (*e.g.*, Lewis structures) and an increase in core 1/3 *O*-glycans that was not seen in our human PDAC analysis. In recent work, Drake and coworkers found increasing levels of extensive fucosylation in PDAC using imaging MS (4), consistent with other literature on terminal fucosylation in pancreatic cancer (40). This is the opposite of what we observe in the KC mouse and points to the importance of cross-referencing the mouse and human data in the modeling of early events.

Our analysis pointed to α-2,6-sialylation and its underlying enzyme ST6GAL1 as a potentially important glycan epitope in early pancreatic cancer based on both mouse and human data. Cell culture models of pancreatic cancer and other solid tumors have demonstrated that ST6GAL1 promotes chemoresistance, cell growth, and a stem cell–like phenotype (8, 9, 41, 42, 43, 44). Several of these works found that sialylation of receptors by ST6GAL1 alters their signaling properties (8, 42, 43). In recent work, sialylation by ST6GAL1 of the oncogenic receptor Erb2 was found to mask the epitope of an anticancer antibody, promoting resistance to treatment (45). Our data reveal the upregulation of ST6GAL1 expression and α-2,6-sialylation in cancerous ducts. Recent imaging MS shows that this epitope is more specific to primary PDAC (4). Our deletion of ST6GAL1 in only pancreatic-specific lineages in the KC mouse (ST6KC) enabled the assessment of the ductal intrinsic impact of ST6GAL1 in promoting cancer formation and progression. In ST6KC, we observed significant protection against the formation of PanIN lesions, an early step in malignant transformation, and a reduction of fibrosis, which is associated with immune suppression and resistance to treatment in patients with PDAC (46). This is consistent with the ductal-specific upregulation observed in human samples and early induction of this enzyme in the KC mouse. Overall, histological assessment of our ST6KC mice demonstrates a clear protective role for genetic deletion of ST6GAL1 in PDAC. Our work highlights the importance of identifying glycans whose expression matches between human patients and the mouse models to enable the study of glycans in cancer.

## Data Availability

All lectin microarray data are available on Synapse (https://doi.org/10.7303/syn22727017). All transcriptomic data are from publicly available sources cited within the article.

## Supplemental data

This article contains supplemental data.

## Conflict of interest

The authors declare no competing interests.
