# Supplementary material for: Integrated Systems Analysis of the Murine and Human Pancreatic Cancer Glycomes Reveals a Tumor-Promoting Role for ST6GAL1
Source: Mol Cell Proteomics. 2021 Oct 9;20:100160. doi: 10.1016/j.mcpro.2021.100160 (PMC8604807; doi:10.1016/j.mcpro.2021.100160)
Supplement: Supplemental Figures S1–S9 and Tables S1–S3 [file mmc1.docx]

**Supplemental Materials**

**Integrated Systems-Analysis of the Murine and Human Pancreatic Cancer Glycomes Reveal a Tumor Promoting Role for ST6GAL1**

Emma Kurz^1‡^, Shuhui Chen^2‡^, Emily Vucic^3^*,* Gillian Baptiste^4^, Cynthia Loomis^5^*,* Praveen Agrawal^4^, Cristina Hajdu^4^*,* Dafna Bar-Sagi^3*^, and Lara K. Mahal^2,6*^

^1^ Department of Cell Biology, NYU Grossman School of Medicine, New York, NY

^2^ Biomedical Research Institute, Department of Chemistry, New York University, New York, NY

^3^ Department of Biochemistry and Molecular Pharmacology, NYU Grossman School of Medicine, New York, NY

^4^ Department of Pathology, NYU Grossman School of Medicine, New York, NY

^5^ Office of Science and Research, NYU Grossman School of Medicine, New York, NY

^6^ Current Address: Department of Chemistry, University of Alberta, Edmonton, AB, Canada

‡Authors Contributed Equally

*Corresponding authors: [lkmahal@ualberta.ca](mailto:lkmahal@ualberta.ca) ; [Dafna.Bar-Sagi@nyulangone.org](mailto:Dafna.Bar-Sagi@nyulangone.org)

**Table of Contents**

**Supplemental Figure S1.** Glycomic analysis of male and female KC mice at 14 weeks of life

**Supplemental Figure S2**. Glycomic analysis of female KC mice at 14 weeks of life

**Supplemental Figure S3**. Glycomic analysis of male KC mice at 14 weeks of life

**Supplemental Figure S4.** Time-course study of male KC mice

**Supplemental Figure S5**. Glycomic analysis of human PDAC samples

**Supplemental Figure S6**. Corroboration of tumor and normal ductal compartments by markers in single cell sequencing

**Supplemental Figure S7**. Elevated expression of ST6GAL1 is associated with poor prognosis

**Supplemental Figure S8**. Single cell sequencing analysis of select glycogenes

**Supplemental Figure S9**. Human TMA Quantification of SNA and ST6GAL1 Cell Type Specific Staining

**Supplemental Table 1**. Patient Characteristics for Samples Used in Human Lectin Microarray

**Supplemental Table 2**. Lectin Microarray Information

**Supplemental Table 3**. Lectins used in Human and Mouse Lectin microarrays


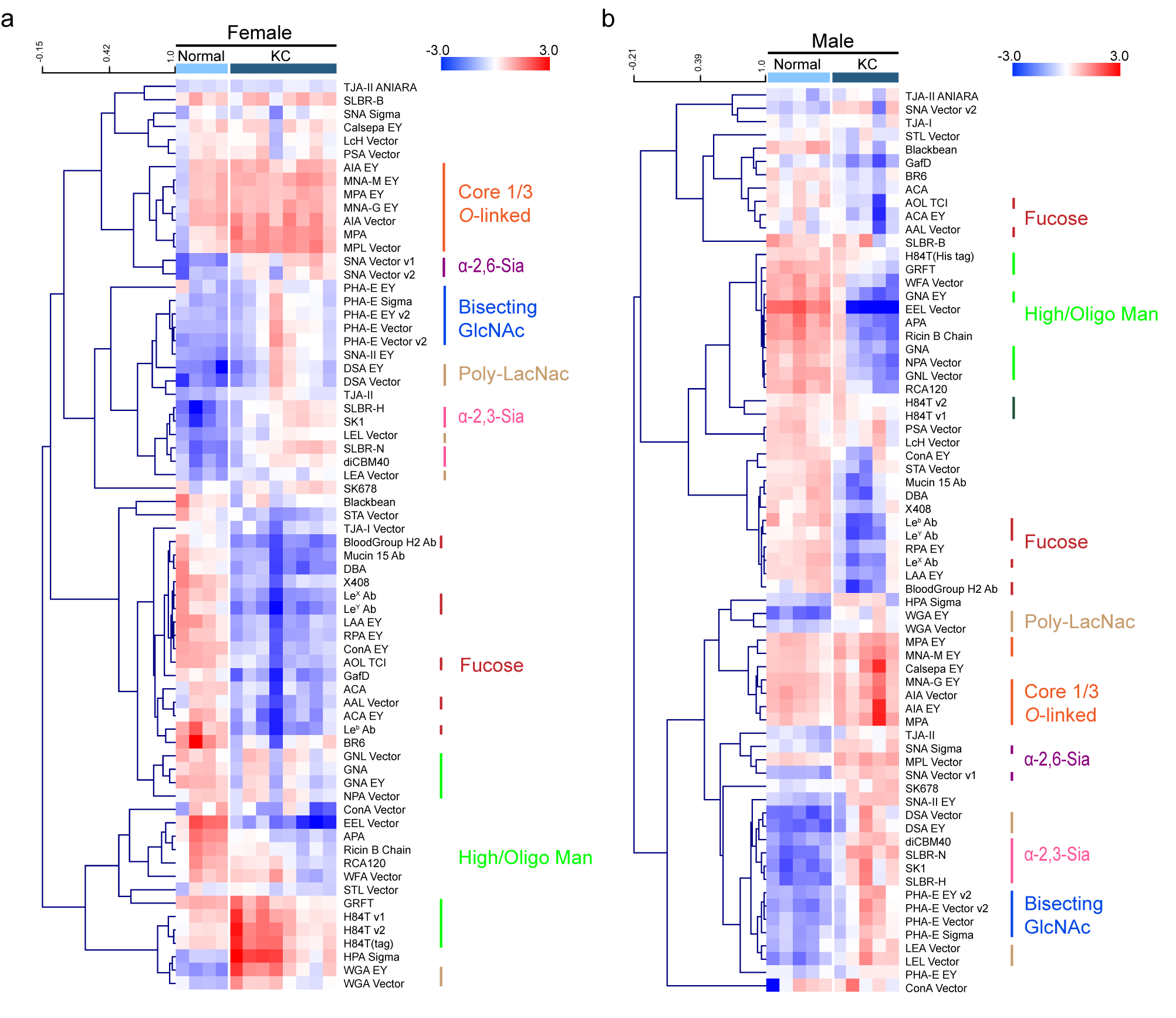


**Supplementary Figure S1. Glycomic analysis of male and female KC mice at 14 weeks of life.** a) Heatmap of female mice lectin microarray data with the complete list of lectins. Median normalized log_2_ ratios (Sample (S)/Reference(R)) were ordered by sample type (Normal, n = 4; KC, n = 8). Red, log_2_(S) > log_2_(R); blue, log_2_(R) > log_2_(S). Lectins binding $\alpha$-2,3-sialosides (pink), $\alpha$-2,6-sialosides (purple), bisecting GlcNAc (navy), poly-*N*-Acetyl-D-Lactosamine (poly-LacNac, brown), high- and oligo-mannose (bright green), core 1/3 *O*-linked glycans (orange), and fucose (red) are highlighted to the right of the heatmap. b) Heatmap of male mice lectin microarray data with the complete list of lectins. Median normalized log_2_ ratios (Sample (S)/Reference(R)) were ordered by sample type (Normal, n = 5; KC, n = 5). Red, log_2_(S) > log_2_(R); blue, log_2_(R) > log_2_(S). Lectins binding $\alpha$-2,3-sialosides (pink), $\alpha$-2,6-sialosides (purple), bisecting GlcNAc (navy), poly-*N*-Acetyl-D-Lactosamine (brown), oligo-mannose (bright green), high-mannose (forest green), core 1/3 *O*-linked glycans (orange), and fucose (red) are highlighted to the right of the heatmap.


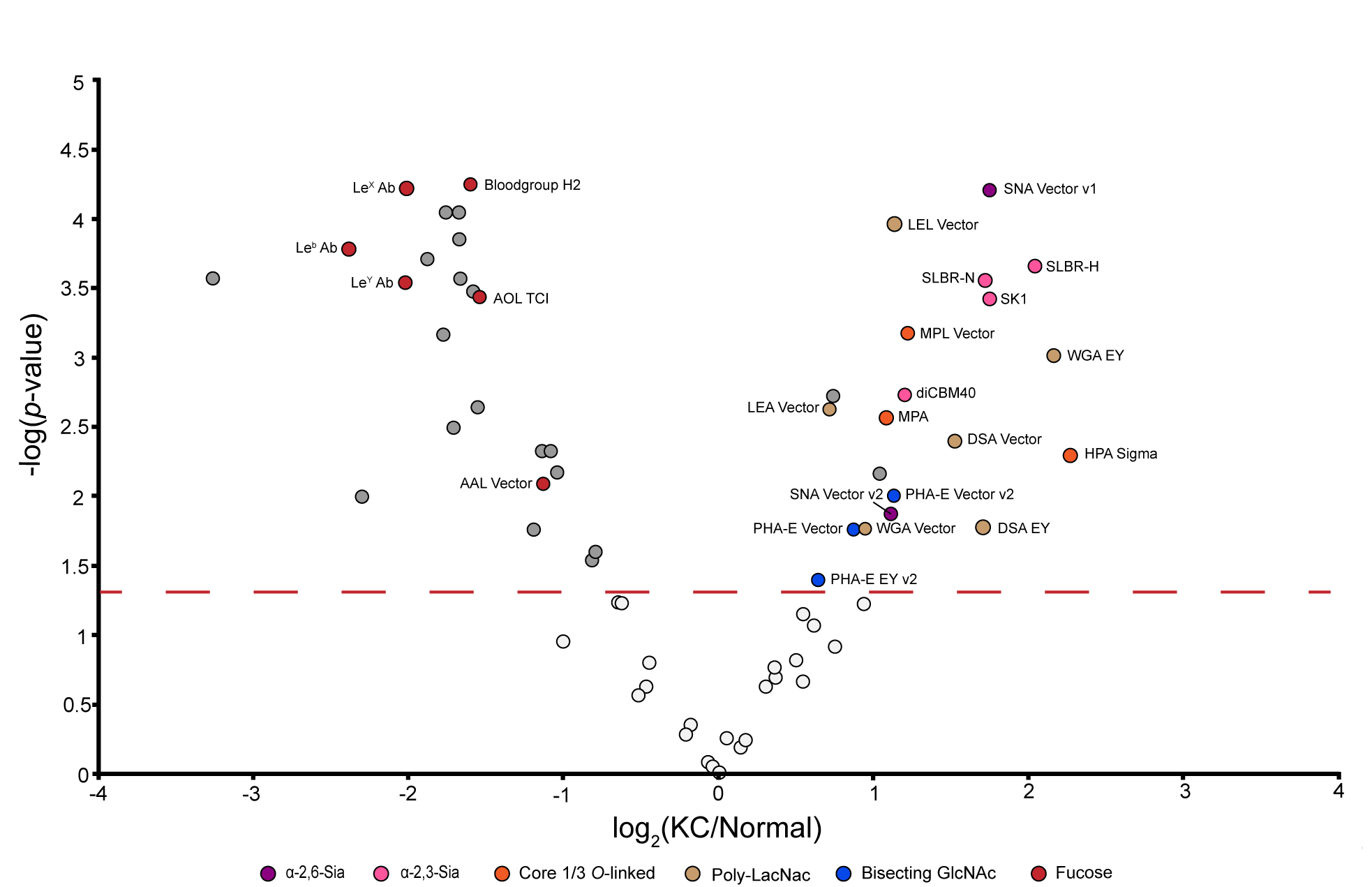


**Supplemental Figure S2. Glycomic analysis of female KC mice at 14 weeks of life.** Volcano plot analysis showed a decrease in fucose levels in female KC mice compared to normal samples (left panel). KC samples showed an increase in $\alpha$-2,6-sialosides, $\alpha$-2,3-sialosides, bisecting GlcNAc, N-Acetyl-D-Lactosamine (LacNAc) and *O*-linked glycans (right panel). Spot colors correspond to lectin specificity; the dotted line represents a significance cutoff of *p*-value ≤ 0.05 across the mice samples.


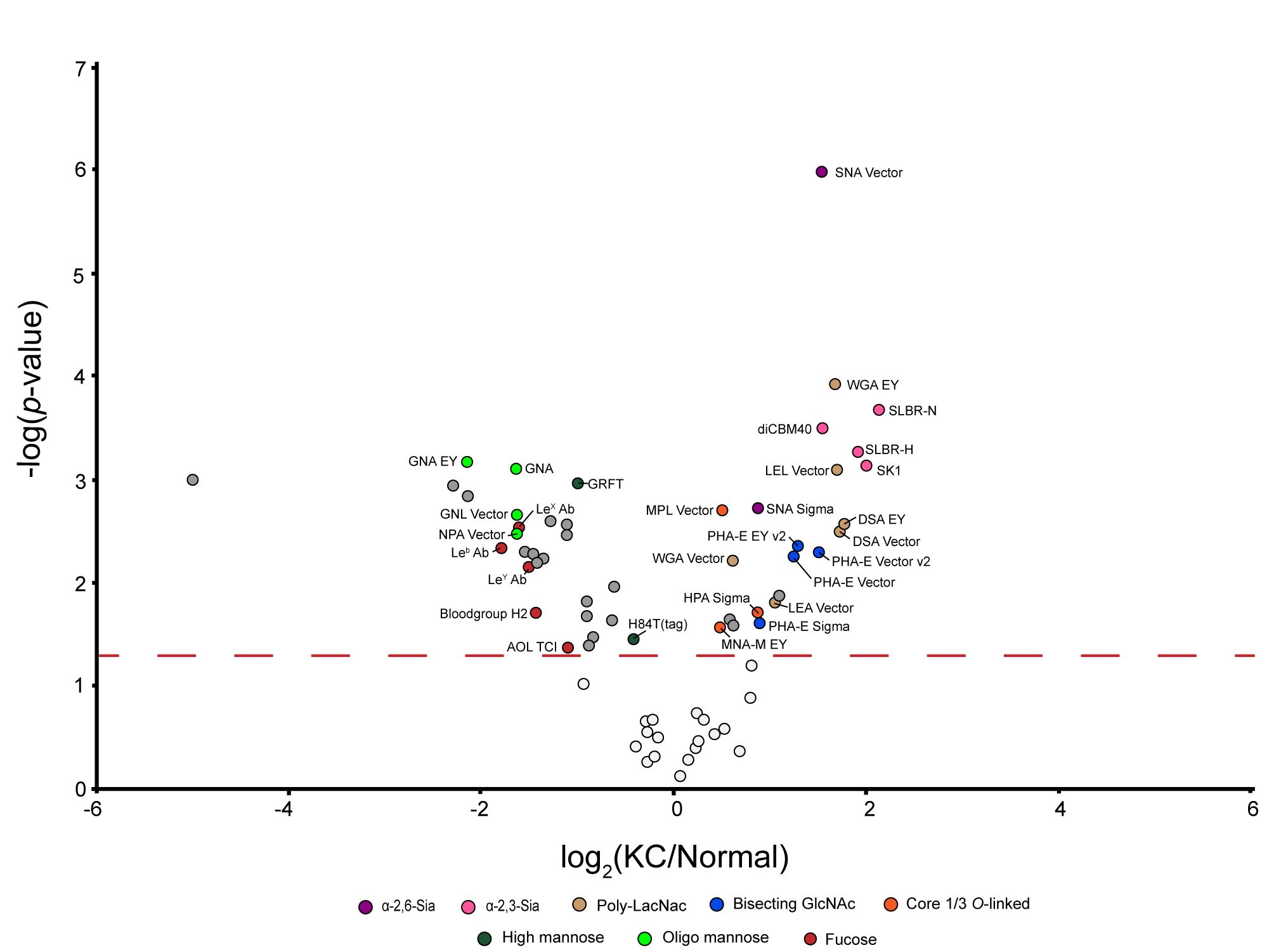


**Supplemental Figure S3. Glycomic analysis of male KC mice at 14 weeks of life.** Volcano plot analysis showed a decrease in oligo-mannose, high-mannose, and fucose levels in male KC mice compared to normal samples (left panel). KC samples showed an increase in $\alpha$-2,6-sialosides, $\alpha$-2,3-sialosides, bisecting GlcNAc, N-Acetyl-D-Lactosamine (LacNAc) and O-linked glycans (right panel). Spot colors correspond to lectin specificity; the dotted line represents a significance cutoff of p-value ≤ 0.05 across the mice samples.


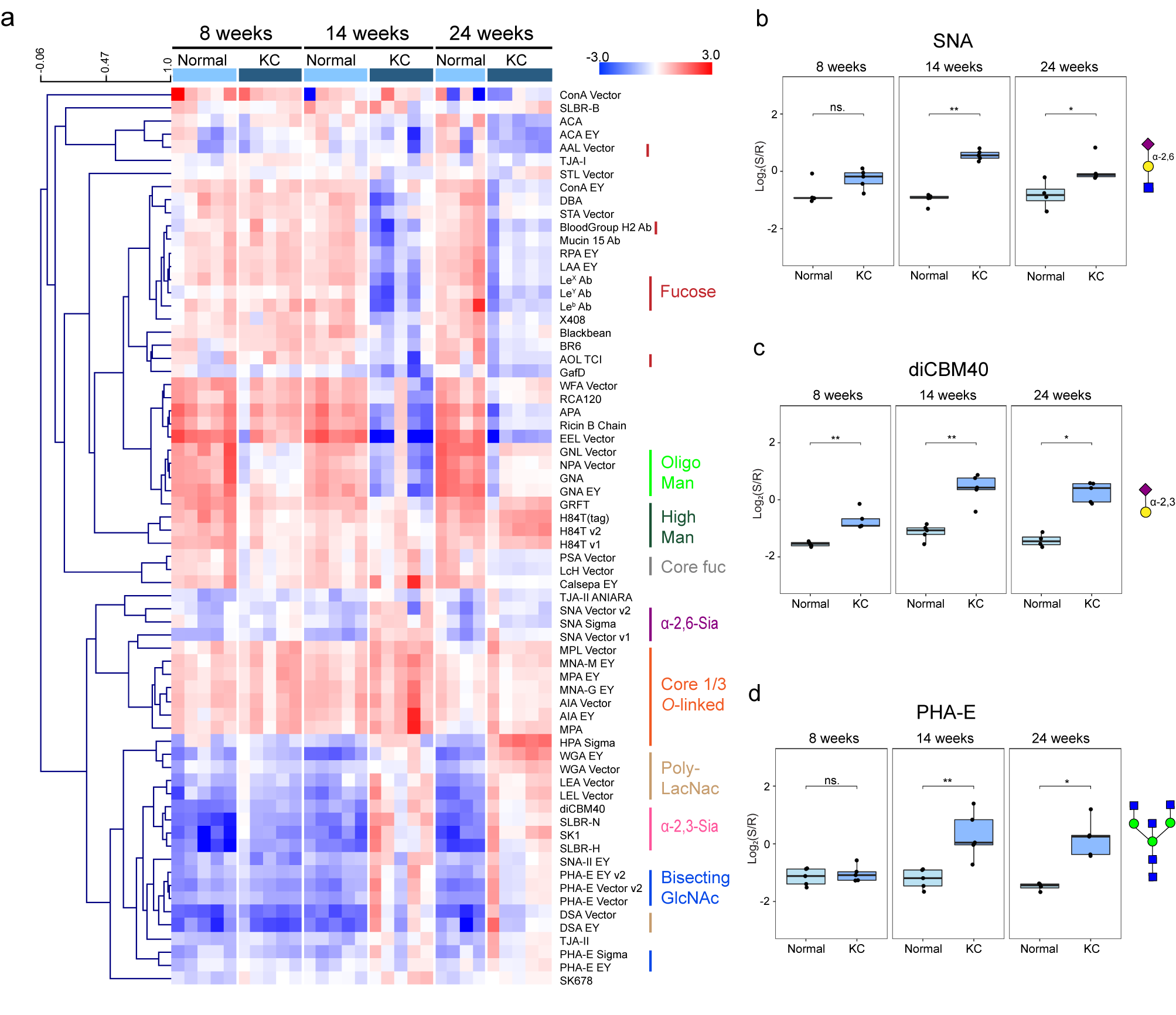


**Supplemental Figure S4. Time-course study of male KC mice.** a) Heat map of lectin microarray data. Median normalized log_2_ ratios (Sample (S)/Reference(R)) of pancreatic samples were ordered by timepoints (8 weeks: Normal, n = 5, KC, n = 5; 14 weeks: Normal, n = 5, KC, n = 5; 24 weeks: Normal, n = 4, KC, n = 5). Red, log_2_(S) > log_2_(R); blue, log_2_(R) > log_2_(S). Lectins binding $\alpha$-2,3-sialosides (pink), $\alpha$-2,6-sialosides (purple), bisecting GlcNAc (navy), N-Acetyl-D-Lactosamine (brown), Oligo-mannose (bright green), high-mannose (forest green), core fucose(charcoal), *O*-linked glycans (orange), and fucose (red) are highlighted to the right of the heatmap. b) Time-course analysis of $\alpha$-2,6-sialosides by SNA. c) Time-course analysis of $\alpha$-2,3-sialosides by diCBM40. d) Time-course analysis of bisecting GlcNAc by PHA-E as a function of time points. Normal samples: light blue; KC samples: dark blue. ns.: Not statistical; *: *p*<0.05; **: *p*<0.01. Wilcoxon’s t-test. Glycans bound by lectins are shown in the Symbolic Nomenclature for Glycomics (SNFG) at the side of the boxplots.

**
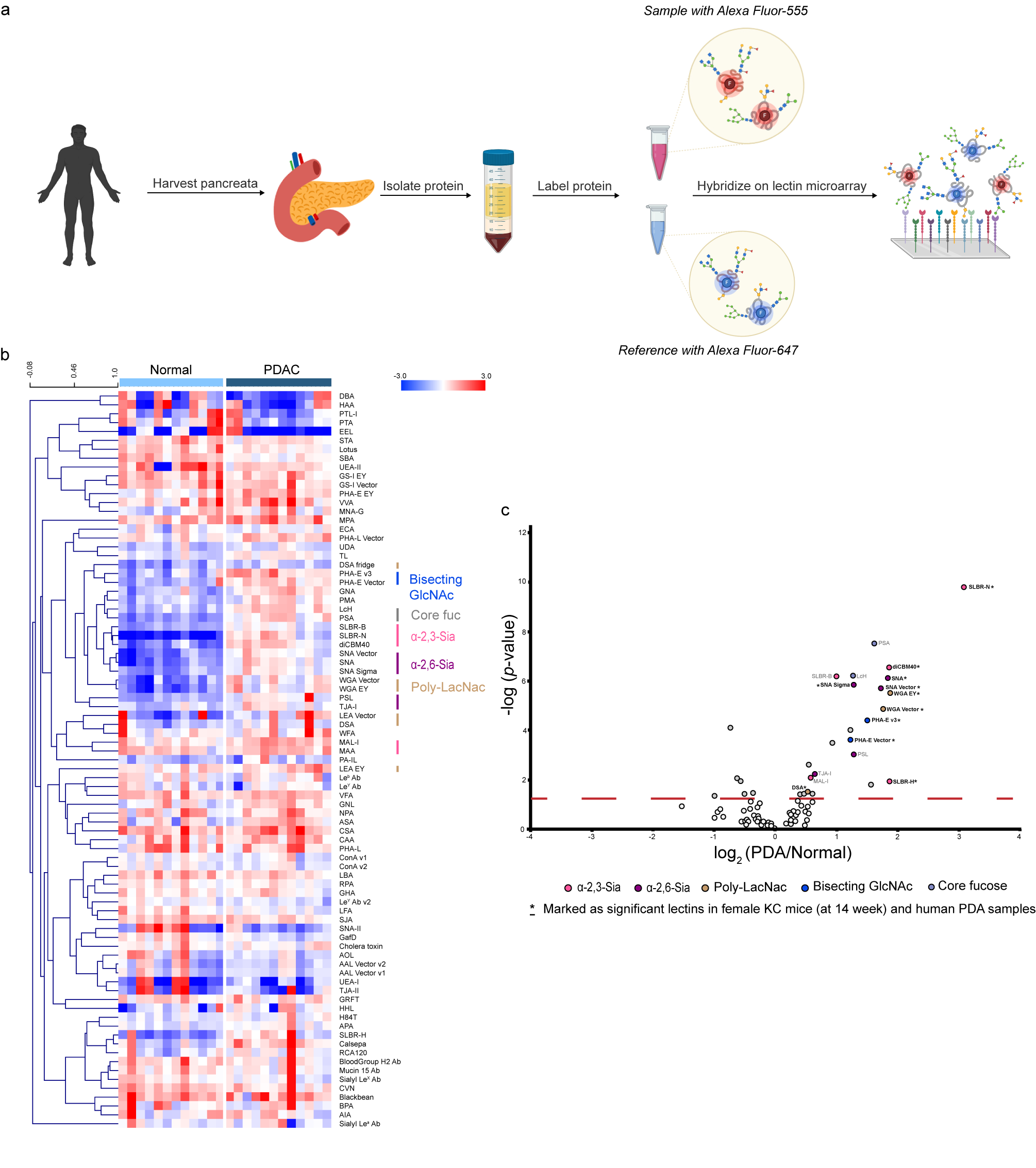
**

**Supplemental Figure S5. Glycomic analysis of human PDAC samples.** a) Workflow of sample preparations for dual-color lectin microarray analysis. Glycoproteins were isolated from pancreata and labeled with Alexa Fluor 555-NHS. A pooled reference was orthogonally labeled with Alexa Fluor 647-NHS. Equal amounts of sample and reference were mixed and hybridized on lectin microarrays (>100 probes). b) Heatmap of human lectin microarray data present with the complete list of lectins. Median normalized log_2_ ratios (Sample (S)/Reference(R)) were ordered by sample type (Normal, n = 12; PDAC, n = 12). Red, log_2_(S) > log_2_(R); blue, log_2_(R) > log_2_(S). Lectins binding α-2,3-sialosides (pink), α-2,6-sialosides (purple), bisecting GlcNAc (navy), poly-*N*-Acetyl-D-Lactosamine (poly-LacNAc, brown), and core fucose (charcoal) are highlighted to the right of the heatmap. b) Volcano plot analysis showed an increase in α-2,3-sialosides (pink), α-2,6-sialosides (purple), bisecting GlcNAc (navy), poly-*N*-Acetyl-D-Lactosamine (poly-LacNAc, brown) and core fucose (slategrey) (on the right panel). Significant lectins (*p*< 0.05) showed in both human PDAC samples and female KC mice samples at 14 weeks were bold and marked as asterisk (*). Spot colors correspond to lectin specificity; the dotted line represents a significance cutoff of *p*-value ≤ 0.05 across the biological samples.

**
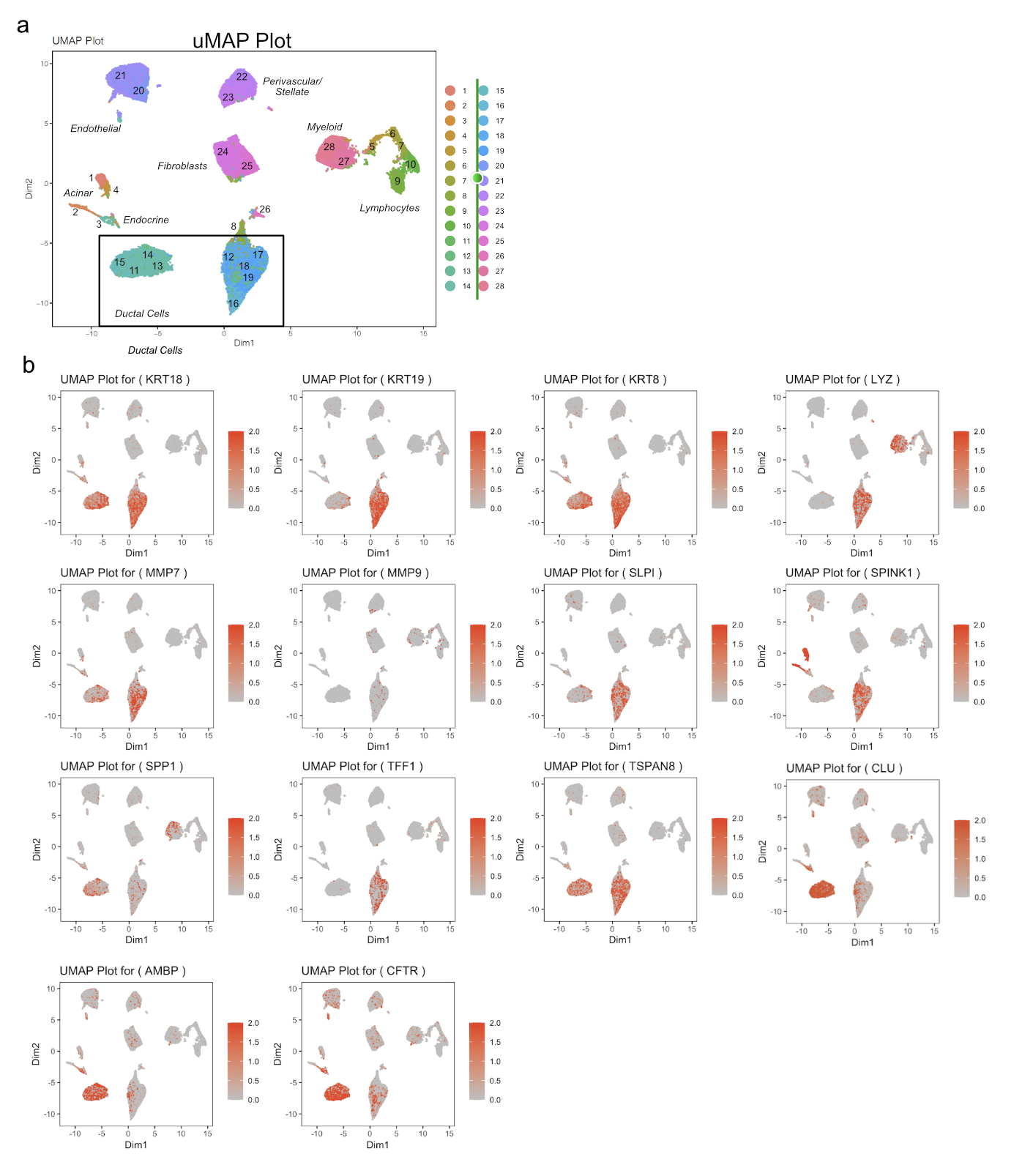
Supplemental Figure S6. Corroboration of tumor and normal ductal compartments by markers in single cell sequencing.** a) uMAP plot representing all cells isolated from PDAC (n=24) patients and normal pancreata (n=11) pooled on single cell-sequencing and colored and numbered by cluster. b) uMAP plots representing expression of a panel of typical genes that identify cells of ductal origin, including: KRT18, KRT19, KRT8, LYZ, MMP7, MMP9, SLPI, SPINK1, SPP1, TFF1, TSPAN8, CLU, AMBP, and CFTR to validate the identity of ductal clusters in normal and PDAC samples.


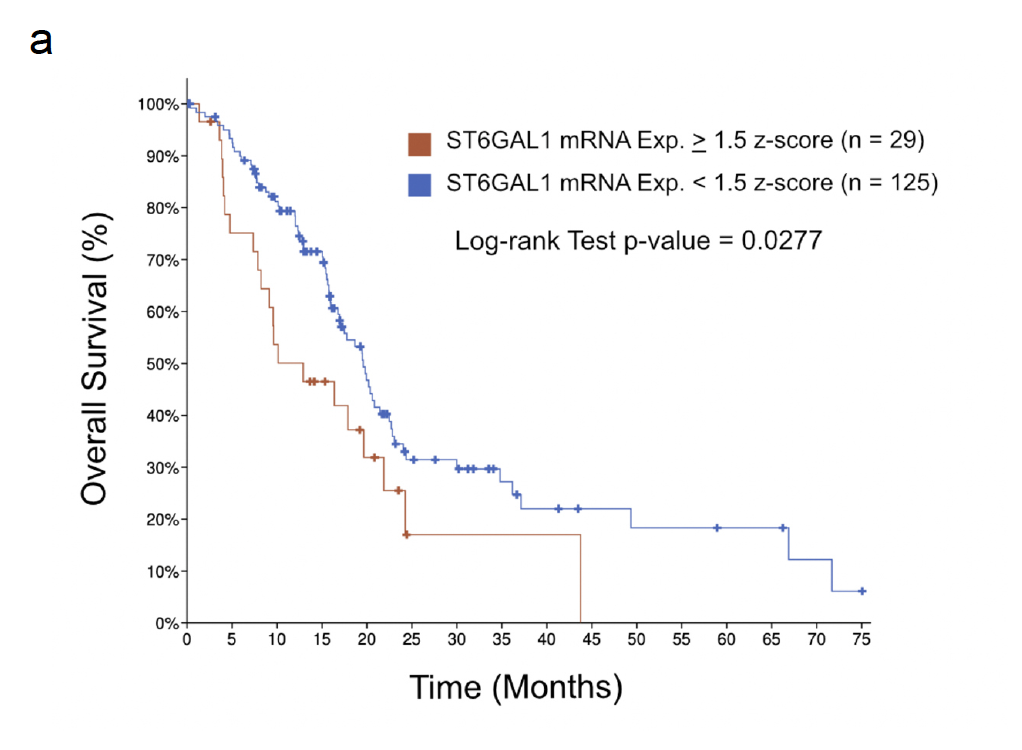


**Supplemental Figure S7. Elevated expression of ST6GAL1 is associated with poor prognosis.**

a) Survival analysis of (n=154) verified human PDAC tumors from TCGA Pan-Cancer Clinical Data Resource (TCGA-CDR) processed in cBioPortal and separated by relative mRNA z-score for ST6GAL1 expression. (Log-rank Test p-value < 0.05 = statistical significance).

**Supplementary Figure S8.** uMAP plots representing cells isolated from PDAC (n=24) patients and normal pancreata (n=11) pooled on single cell-sequencing and colored in red gene expression. Data for all glycogenes analyzed in Figure 4b are shown. Significantly higher expression in tumor ducts was observed for MGAT3, ST3GAL4, ST6GAL1, FUT8, and B3GNT3 (Student’s t-test (two-tailed), *p* < 0.05.
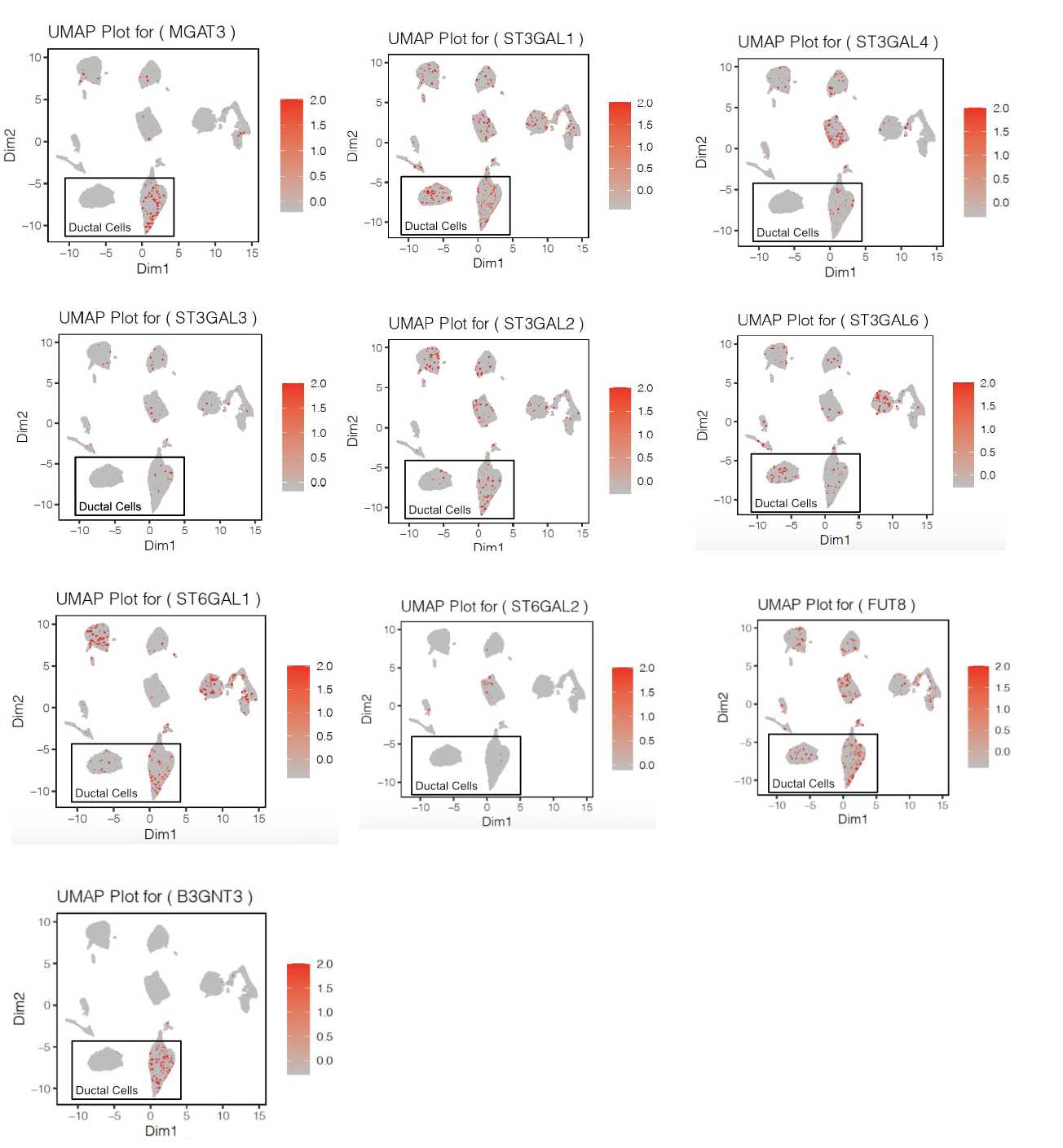


**
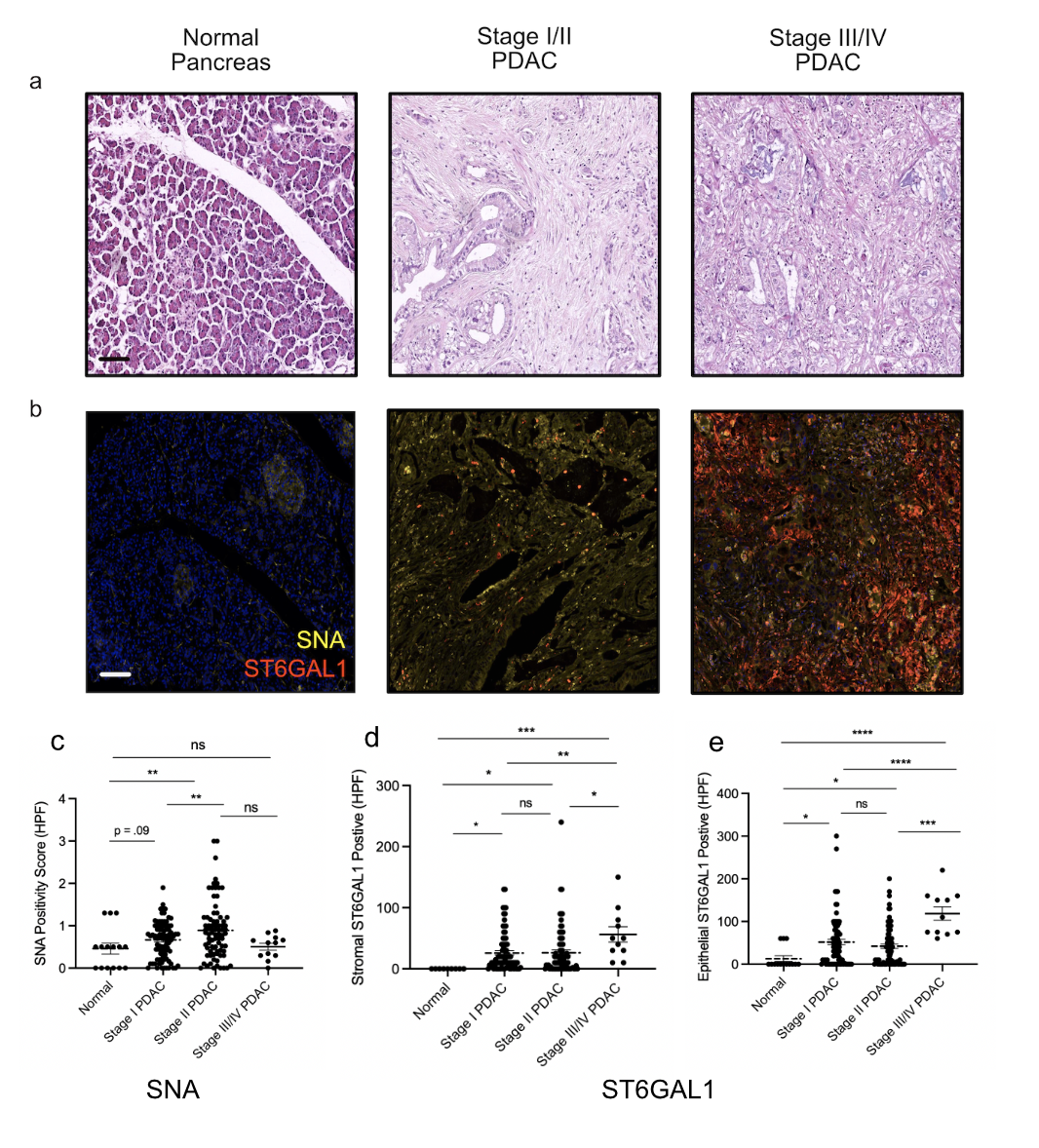
Supplemental Figure S9. Human TMA Quantification of SNA and ST6GAL1 Cell Type Specific Staining.** a) H&E of normal pancreas (left), stage I pancreatic adenocarcinoma (center), and stage IV PDAC (right) stained from a BioMax human tissue microarray. b) Multiplex OPAL IF staining of SNA (yellow), ST6GAL1 (red) and DAPI (blue) on corresponding normal pancreas, Stage I, and Stage IV pancreatic adenocarcinoma from BioMax human tissue microarray. Scale bars represent 100μm. c) Quantification of SNA positive cells per high powered field based on multiplex IF in normal pancreas compared to tumor samples at each stage of human PDAC. d) Quantification of ST6GAL1 positivity in non-epithelial stromal cells in normal pancreas compared to tumor samples at each stage of human PDAC e) Quantification of ST6GAL1 positivity in epithelial cells in normal pancreas compared to tumor samples at each stage of human PDAC (ns: p > 0.05; *: p < 0.05; **: p < 0.01; ***: p < 0.001; ****: p < 0.0001).

**Supplemental Table 1. Patient Characteristics for Samples Used in Human Lectin Microarray**

| **Tissue ID** | **PDAC^[i]^/ Normal** | **Age** | **M/F^[ii]^** | **Race/Ethnicity** | **Stage^[iii]^** | **Histology (Grade)^[iv]^** |
| --- | --- | --- | --- | --- | --- | --- |
| 420 3I-2/1 | PDAC | 68 | M | African American | pT2, N2 | Moderately/Poorly, G3 |
| 495 1D-1/2 | PDAC | 75 | M | Asian | pT3, N1 | Moderately, G2 |
| 182 3O-1/2 | PDAC | 63 | M | White | pT2, N1 | Adeno with Focal Squamous, G3 |
| 741 2N | PDAC | 77 | F | Other | pT2, N2 | Moderately, G2 |
| 321 1U-1/2 | PDAC | 73 | M | Other | pT2, N1 | Poorly, G3 |
| 6124 3X | PDAC | 70 | F | White | pT2, N1 | Poorly, G3 |
| 8750 2AG | PDAC | 81 | F | White | pT2, N0 | Poorly, G3 |
| 7098 4Y | PDAC | 68 | M | White | pT1, N2 | Moderately, G2 |
| 2741 3AF | PDAC | 89 | F | White | pT2, N1 | Poorly, G3 |
| 700 1AM-1/2 | PDAC | 64 | M | White | pT2, N1 | Moderately, G2 |
| 307 1A-1/2 | Normal | 78 | M | White | N/A | N/A |
| 942 1V-1/2 | Normal | 82 | F | White | N/A | N/A |
| 823 1B-1/2 | Normal | 62 | F | African American | N/A | N/A |
| 756 1A-1/2 | Normal | 63 | F | White | N/A | N/A |
| 411 1C-1/2 | Normal | 63 | M | White | N/A | N/A |
| 065 1W-1/2 | Normal | 40 | M | African American | N/A | N/A |
| 5496 1J | Normal | 76 | F | White | N/A | N/A |
| 9942 1V | Normal | 82 | F | White | N/A | N/A |
| 2233 1Q | Normal | 66 | F | White | N/A | N/A |
| 5756 1A | Normal | 63 | F | White | N/A | N/A |
| 9306 2AE | Normal | 50 | F | Other | N/A | N/A |

^[i]^ PDAC = Pancreatic Ductal Adenocarcinoma

^[ii]^ M = Male, F = Female

^[iii]^ pT1 = primary tumor limited to pancreas, excision complete with free margins; pT2 = primary tissue extends beyond pancreas, excision complete with free margins; pT3 = tumor invades beyond pancreas, excision complete with microscopic residual tumor/macroscopic residual tumor or adjacent malignant effusion. N1 = regional lymph node metastasis; N2 = distant lymph node metastasis.

^[iv]^ G = Tumor histological grade: G2 = intermediate grade, moderately differentiated, cells somewhat abnormal; G3 = high grade, poorly differentiated, cells very abnormal

**Supplemental Table 2. Lectin Microarray Information**

|  | | | **Description** |
| --- | --- | --- | --- |
| 1. **Sample: Glycan-containing sample (e.g. glycan, glycoprotein, cell lysate etc.)** | | | |
| Description of Sample | Glycoproteins extracted from formalin-fixed paraffin-embedded (FFPE) tissues from human PDAC patients and KC mouse samples | | |
| Sample preparation protocol | Both human and mouse samples were fixed in 10% neutral buffered formalin. For analysis of tumor samples, hematoxylin and eosin-stained slides were reviewed to ensure the size of tumor. Unstained cut sections were mounted on the slide and macro-dissected to remove containing normal cells. 2 ⋅ 20⎧m sections of each FFPE tissue were scraped into a 1.5mL microcentrifuge tube. 1mL xylene and 200⎧L 100% ethanol were added to the microcentrifuge tube, incubated for 10 mins at room temperature, centrifuged at 14,000 ⋅ g for 3 mins, and supernatant was removed; repeat twice. The deparaffinized tissue were rehydrated with a graded series of ethanol. 1mL 100% ethanol was added to each tissue, incubated for 10 min at room temperature, centrifuged at 14,000 ⋅ g for 3 mins, and supernatant was removed. Then 1mL 90% ethanol was added to each tissue, incubated for 10 min at room temperature, centrifuged at 14,000 ⋅ g for 3 mins, and supernatant was removed. Next, 1mL 70% ethanol was added to each tissue, incubated for 10 min at room temperature, centrifuged at 14,000 ⋅ g for 3 mins, and supernatant was removed. Tissue pellet was heated at 90°C for 30 seconds to remove any remaining ethanol, and allowed to dry at room temperature. The rehydrated tissue was suspended in 200⎧L 10mM sodium citrate buffer (pH 6.0) and incubated at 95°C for 1 hour. The tissue was centrifuged at 14,000 ⋅ g for 5 mins, and supernatant was removed. The tissue was washed with 1⋅ PBS (pH 7.4). The pellet was solubilized with 100⎧L PBS containing 0.5% Nonidet P-40 (NP40). Samples were sonicated (70% power, 1 min), and incubated on ice for 30 mins; repeat one more time. Sample was centrifuged at 14,000 ⋅ g for 15 mins at 4°C, and supernatant was collected for glycomic analysis. | | |
| Labeling protocol for sample detection | Samples are labelled with Alexa Fluor 555-NHS (Thermo Fisher). | | |
| Two-color reference (if used) | A pooled reference samples are labelled with Alexa Fluor 647-NHS (Thermo Fisher). | | |
| Assay protocol | Lectin microarrays are blocked with blocking buffer for one hour at room temperature. Slides are rinsed twice with PBST (0.005%) and once with PBS, then dry the slide using a slide spinner. Each slide was mounted on a 24-well format hybridization cassette (Arrayit), in which each well contains a subarray. To each well, add equal amounts of samples and universal reference, and dilute with PBS and PBST (0.2%) to reach the final volume (150uL). Incubate the slides on an orbital shaker for two hours at room temperature in the dark. After hybridization, wash the arrays with PBST (0.005%) twice for ten minutes, and twice for five minutes. Once finished, remove the slides from the cassette, and immerse the slides in ultrapure water, and dry the slides using a slide spinner. | | |
| **2.** **Lectin Library** | | | |
| General description of the lectin library used in the array | Lectin microarrays are generated in house. | | |
| List of lectins and glycan binding proteins, source, concentration and buffer | Please see **Supplemental Table 3**. | | |
| Modification of lectins (e.g. biotin) if any. | N/A | | |
| 1. **3.** **Immobilization Surface; e.g., Microarray Slide** | | | |
| Immobilization surface | Nexterion Slide H Barcoded 3D Hydrogel Coated | | |
| Manufacturer | Schott North America | | |
| Custom preparation of surface | N/A | | |
| **4. Array Production** | | | |
| Description of Arrayer | Nano-Plotter 2.1 piezoelectric printer (GeSim, Germany) with cooled microwell plate holder and cooled printing deck | | |
| Lectin deposition | Three replicates of each lectin are printed onto each subarray. | | |
| Printing conditions | Dilute lectins to the pre-determined concentrations in the print buffer (final concentration of print buffer: 0.01% Tween-20, 1mM monosaccharide in PBS; Please see **Supplemental** **Table 3** for the concentrations of lectins). Load the mixed solution to the microplate. Before printing, check the humidity of the print chamber. The humidity should be kept around 50% during the entire printing. Ensure both microwell plate holder and printing deck are cooled. Adjust the cooling temperature based on ambient temperature and the temperature of the cooled slide deck surface, preventing moisture building up inside the print chamber. Once printing is complete, allow the slides to dry for at least one hour. | | |
| Array layout | | For each microarray, it contains 24 subarrays (3 columns and 8 rows). In each subarray, triplicates of a lectin are printed, and for a row with five lectins, the spot layout should be 15 columns. The row number depends on how many lectin probes are printed on the arrays (i.e., 110 lectins require 22 rows). | |
| Quality control | | Well-characterized glycoproteins including fetuin, asialofetuin, RNase B and bovine mucin are used for quality control of the printed microarrays. | |
| 1. **5. Detector and Data Processing** | | | |
| Instrument (scanner, flow cytometer) | Fluorescent Slide Scanner Genepix 4300A (Molecular Devices) | | |
| Instrument settings | Preview the slide to adjust photomultiplier gain (PMT) for each channel (Alexa Fluor-555: 532nm, Alexa Fluor-647: 635nm) so that the signals are not saturated and within the linear detection range. | | |
| Image analysis software | GenePix Pro 7 (Molecular Devices) | | |
| Data processing and statistical analysis | Extracted data is processed for quality checks using Grubbs outlier test with $\alpha$ = 0.05. Log_2_ values of the average signals are median-normalized over the individual subarray in each channel. | | |
| **6.** **Lectin Microarray Data Presentation** | | | |
| Data presentation and interpretation | Hierarchical clustering of the processed data is performed using Pearson Correlation coefficient, and visualized with Multi-experiment Viewer (MeV, v4.8, TM4 Microarray Software Suite). If a lectin’s SNR (signal-to-noise ratio) < 3 for more than one third of the total samples, then this lectin is considered as inactive and excluded from the list. *P*-values are calculated using nonparametric statistical tests, which are generated by R (v3.6.1). | | |
| **7. Data Location** | | | |
| Data Location | Synapse ID: syn22727017 | | |

**Supplemental Table 3. Lectins used in Human and Mouse Lectin microarrays**

| **Lectin** | **Species/Origin** | **Print Conc.**  **(µg/mL)** | **Rough Specificity /Inhibitory monosaccharide** | **Vendor/Source** |  |
| --- | --- | --- | --- | --- | --- |
|  |  |  |  |  |  |
| AAL ^a, b^ | *Aleuria aurantia* | 1000 | Fucose | Vector |  |
| ACA ^a, b^ | *Amaranthus Caudatus* | 1000 | Gal-β1,3-GalNAc | Vector |  |
| AIA ^a, b^ | *Artocarpus integrifolia* | 500 | β1,3-GalNAc | Vector/EY |  |
| AMA ^a, b^ | *Allium moly* | 500 | Oligo mannose | EY |  |
| Anti-B.G.H2 ^a, b^ | MAb mouse IgM [A46-B/B10] | undiluted | Blood group H2 antigen | Santa Cruz Biotechnology |  |
| Anti-Forssman ^a^ | MAb Rat IgM [117C9] | undiluted | Forssman Antigen | Abcam |  |
| Anti-Lewis B ^a, b^ | IgM [T218] | undiluted | Lewis B | Sigma |  |
| Anti-Lewis X ^a, b^ | MAb mouse IgM [P12] | undiluted | Lewis X | Abcam |  |
| Anti-Lewis Y ^a, b^ | MAb mouse IgM [F3] | undiluted | Lewis Y | Abcam |  |
| Anti-MUC5AC human ^a, b^ | Mab mouse IgG1 [CLH2] | undiluted | human MUC5AC | Sigma |  |
| Anti-MUC5AC mouse ^a^ | Goat polyclonal to mouse MUC5AC | undiluted | mouse MUC5AC | LSBio |  |
| Anti-Mucin 15 ^a, b^ | Mab mouse IgG1 [H-5] | undiluted | Mucin 15 | Santa Cruz Biotechnology |  |
| Anti-Sialyl Lewis A ^a^ | Mab mouse IgG1 | undiluted | Sialyl Lewis A | Abcam |  |
| Anti-Sialyl Lewis X ^a^ | Mab mouse IgM | undiluted | Sialyl Lewis X | Abcam |  |
| AOL ^a, b^ | *Aspergillus oryzae* | 1000 | Fucose | TCI America |  |
| APA ^a, b^ | *Abrus precatorius* | 500 | Gal-β1,3-GalNAc / Lac | EY |  |
| ASA ^a, b^ | *Allium sativum* | 1000 | Mannose | EY |  |
| Blackbean ^a, b^ | *Blackbean crude* | 1000 | GalNAc | EY |  |
| BPA ^a, b^ | *Bauhinia purpurea* | 500 | β-Gal / β-GalNAc | Vector |  |
| BR6 ^b^ | unknown (from unpublished work) | 480 | under investigation | Gift from Dr. Barbara Bensing |  |
| CA ^b^ | *Colchicum autumnale* | 1200 | Bi-antennary N-linked glycans | EY |  |
| CAA ^a^ | *Caragana arborescens* | 1000 | Bi-antennary N-linked glycans | EY |  |
| Calsepa ^a, b^ | *Calystegia sepium* | 1000 | Bisecting N-linked glycans | EY |  |
| CCA ^a^ | *Cancer antennarius* | 1000 | 9-O-Acetly sialylation / 4-O-Acetyl sialylation | EY |  |
| Cholera Toxin ^a, b^ | *Vibrio cholerae* | 1000 | GM1 ganglioside | Sigma |  |
| Con A ^a, b^ | *Canavalia ensiformis* | 1000 | Tri-mannose core | EY/Vector |  |
| CSA ^a, b^ | *Cystisus scoparius* | 1000 | Terminal GalNAc | EY |  |
| DBA ^a, b^ | *Dolichos Biflorus* | 1000 | GalNAc | Vector |  |
| diCBM40 ^a, b^ | engineered NanI from *Clostridium perfringens* | 1000 | α Sialylation | Generated in house |  |
| DSA ^a, b^ | *Datura stramonium* | 500 | LacNAc | EY/Vector |  |
| ECA ^a, b^ | *Erythrina cristagalli* | 1000 | LacNAc | Vector |  |
| EEL/EEA ^a, b^ | *Eunonymus europaeus* | 1000 | Blood Group B | Vector/EY |  |
| GafD ^a, b^ | recombinant GafD from *Escherichia coli* | 1000 | GlcNAc | Generated in house |  |
| GHA ^a^ | *Glechoma hederacea* | 500 | GalNac | EY |  |
| GNA/GNL ^a, b^ | *Galanthus nivalis* | 1500 | Oligo mannose | Vector/EY |  |
| GS-I ^a, b^ | *Griffonia simplicifoia-I* | 1000 | α-Gal / Lac | Vector/EY |  |
| GS-II ^a, b^ | *Griffonia simplicifoia-II* | 1000 | GlcNAc | Vector |  |
| GS-IB4 ^a, b^ | *Griffonia simplicifoia-I, isolectin B4* | 2000 | Gal | Vector |  |
| H84T ^a, b^ | *Banana lectin* | 1000 | High mannose | Gift from Dr. David Markovitz |  |
| HAA ^a, b^ | *Homarus americanus* | 1000 | Terminal GalNAc | EY |  |
| HHL ^a, b^ | *Hippeastrum Hybrid* | 1500 | Oligo/High mannose | Vector |  |
| HPA ^a, b^ | *Helix pomatia* | 1000 | Blood Group A | Sigma/EY |  |
| LAA ^b^ | *Laburnum alpinum* | 900 | GlcNAc | EY |  |
| LBA ^a^ | *Phaseolus lunatus* | 1000 | Blood Group A | EY |  |
| LcH ^a, b^ | *Lens Culinaris* | 1000 | Core Fucose | Vector |  |
| LEA/LEL ^a, b^ | *Lycopersicon esculentum* | 1000 | GlcNAc | Vector/EY |  |
| LFA ^a^ | *Limax flavus* | 500 | α Sialylation | EY |  |
| Lotus ^a, b^ | *Lotus tetragonolobus* | 1000 | Fucose | Vector |  |
| MAA ^a^ | *Maackia amurensis* | 500 | Sialylation/Sulfation | EY |  |
| MAL-I ^a, b^ | *Maackia amurensis-I* | 2000 | Sialylation/Sulfation | Vector |  |
| MAL-II ^a, b^ | *Maackia amurensis-II* | 2000 | Sialylation/Sulfation | Vector |  |
| MNA-G ^a, b^ | *Morus nigra Morniga G* | 1000 | GalNAc | EY |  |
| MNA-M ^b^ | *Morus nigra Morniga M* | 1000 | Oligo mannose / Gal | EY |  |
| MPA/MPL ^a, b^ | *Maclura pomifera* | 1000 | β1,3-GalNAc | Vector |  |
| NPA ^a, b^ | *Narcissus pseudonarcissus* | 1000 | Oligo mannose | Vector |  |
| PA-I ^b^ | *Pseudomonas aeruginosa* | 1000 | Gal | Sigma |  |
| PA-IL ^a^ | *bacteria* | 1000 | GalNAc | Generated in house |  |
| PHA-E ^a, b^ | *Phaseolus vulgaris Erythroagglutinin* | 1000 | Bisecting GlcNAc | Vector/EY/Sigma |  |
| PHA-L ^a, b^ | *Phaseolus vulgaris Leukoagglutinin* | 1000 | β1,6 Branching N-Link glycans | Vector/EY/Roche |  |
| PMA ^a^ | *Polygonatum multiflorum* | 500 | Oligo mannose | EY |  |
| PNA ^a, b^ | *Arachis hyogaea* | 1000 | Gal-β1,3-GalNAc | Vector/EY |  |
| PSA ^a, b^ | *Pisum sativum* | 1000 | Core Fucose | Vector |  |
| PSL ^a^ | *Polyporus squamosus* | 1000 | α2,6 sialylation | EY |  |
| PTA ^a, b^ | *Psophocarpus tetragonolobus* | 500 | Blood Groups | EY |  |
| PTL-I ^a, b^ | *Psophocarpus tetragonolobus-I* | 1500 | Blood Group A | Vector |  |
| PTL-II ^a, b^ | *Psophocarpus tetragonolobus-II* | 1000 | α2 Fucose | Vector |  |
| RCA120 ^a, b^ | *Ricinus Communis Agglutinin I* | 1000 | Gal / Lac | Vector |  |
| rCVN ^a^ | *recombinant Cyanovirin* | 1000 | High mannose | Gift from Dr. Barry O'Keefe |  |
| rGRFT ^a, b^ | *recombinant Griffithsin* | 1000 | High mannose | Gift from Dr. Barry O'Keefe |  |
| Ricin B Chain ^a, b^ | *Ricinus communis* | 1000 | Gal | Vector |  |
| RPA ^a, b^ | *Robinia pseudoacacia* | 500 | Complex N-link glycans | EY |  |
| rSVN ^a^ | *recombinant Scytovirin* | 1000 | High mannose | Gift from Dr. Barry O'Keefe |  |
| SBA ^a, b^ | *Glycine max* | 1000 | LacdiNAc | Vector |  |
| SJA ^a, b^ | *Sophora japonica* | 1000 | LacdiNAc | Vector |  |
| SK1 ^b^ | *Streptococcus sanguinis SK1* | 1800 | α2,3 sialylation | Gift from Dr. Barbara Bensing |  |
| SK678 ^b^ | *Streptococcus sanguinis SK678* | 450 | α2,3 sialylation | Gift from Dr. Barbara Bensing |  |
| SLBR-B ^a, b^ | *Streptococcus gordonii M99* | 1000 | α2,3 sialylation | Gift from Dr. Barbara Bensing |  |
| SLBR-H ^a, b^ | *Streptococcus gordonii DL1* | 2000 | α2,3 sialylation | Gift from Dr. Barbara Bensing |  |
| SLBR-N ^a, b^ | *Streptococcus gordonii UB10712* | 1000 | α2,3 sialylation | Gift from Dr. Barbara Bensing |  |
| SNA ^a, b^ | *Sambucus nigra* | 500/1000 | α2,6 sialylation | Vector/Sigma |  |
| SNA-II ^a, b^ | *Sambucus nigra-II* | 1000 | α2 Fucose /oligo mannose | EY |  |
| STA/STL ^a, b^ | *Solanus tuberosum* | 500 | GlcNAc | Vector |  |
| TJA-I ^a, b^ | *Trichosanthes japonica-I* | 1000 | α2,6 sialylation | TCI |  |
| TJA-II ^a, b^ | *Trichosanthes japonica-II* | 1000 | α2 Fucose | NorthStar Bioproducts/Aniara Diagnostica |  |
| TL ^a, b^ | *Tulipa sp.* | 700 | GlcNAc | EY |  |
| UDA ^a^ | *Urtica dioica* | 1000 | GlcNAc / Oligo mannose | EY |  |
| UEA-I ^a, b^ | *Ulex europaaeus-I* | 1000 | α2 Fucose | Vector |  |
| UEA-II ^a, b^ | *Ulex europaaeus-II* | 2000 | GlcNAc | Vector |  |
| VFA ^a, b^ | *Vicia faba* | 1000 | GlcNAc | EY |  |
| VVA ^a, b^ | *Vicia villosa* | 1000 | Terminal GalNAc | Vector/EY |  |
| VVA(man) ^a, b^ | *Vicia villosa* | 500 | Mannose | Vector/EY |  |
| X408 ^b^ | unknown (from unpublished work) | 1000 | under investigation | Gift from Dr. Barbara Bensing |  |
| WFA ^a, b^ | *Wisteria floribunda* | 1000 | GalNAc-β1,4 | Vector |  |
| WGA ^a, b^ | *Triticum vulgare* | 1000 | GlcNAc | Vector/EY |  |

^a^ : lectins printed in human lectin microarrays

^b^ : lectins printed in mouse lectin microarrays

*Highlighted lectins did not pass QC for those microarrays.*
